# HMGB1 regulates mitochondrial structure and reactive oxygen species balance during the transition from naïve to primed pluripotency

**DOI:** 10.3389/fcell.2026.1807454

**Published:** 2026-05-12

**Authors:** Tatiana Y. Starkova, Sergey V. Ponomartsev, Veniamin S. Fishman, Nariman R. Battulin, Nikolay D. Aksenov, Evgeny I. Bakhmet, Andrey A. Kuzmin, Dmitry S. Bogolyubov, Sergey A. Sinenko, Alexey N. Tomilin

**Affiliations:** 1 Laboratory of Molecular Biology of Stem Cells, Institute of Cytology of the Russian Academy of Sciences, St. Petersburg, Russia; 2 Laboratory of Developmental Genetics, Institute of Cytology and Genetics, SB RAS, Novosibirsk, Russia; 3 Laboratory of Structural and Functional Organization of the Genome, Novosibirsk State University, Novosibirsk, Russia; 4 Flow Cytometry and Sorting Group, Institute of Cytology of the Russian Academy of Sciences, St. Petersburg, Russia; 5 Laboratory of Cell Morphology, Institute of Cytology of the Russian Academy of Sciences, St. Petersburg, Russia

**Keywords:** cell metabolism, differentiation, embryonic stem cells (ESCs), HMGB1, mitochondria, reactive oxygen species

## Abstract

**Introduction:**

Embryo implantation is characterized by the process of naïve-to-primed pluripotency transition in epiblast cells, involving an anabolic boost, mitochondrial remodeling, and increased proliferation. Yet, the molecular mechanisms underlying these extensive changes remain poorly understood. High mobility group box 1 (HMGB1) is a non-histone, redox-sensitive chromatin protein involved in diverse cellular processes, however its role in pluripotency control has not been fully characterized.

**Methods:**

To determine the function of HMGB1 in mouse embryonic stem cells (ESCs), *Hmgb1*-knockout (KO) ESCs were generated using CRISPR/Cas9 system. KO ESCs were analyzed for cell proliferation, cell cycle progression, and apoptosis, as well as for levels of active mitochondria, mitochondrial membrane potential, and reactive oxygen species (ROS) using fluorescent-based reagents and flow cytometry. Pluripotency was assessed by analyzing the expression of pluripotency markers with immunocytochemistry, Western blotting, qRT-PCR, as well as by teratoma formation assay. Naïve-to-primed pluripotency transition was investigated by *in vitro* culture. Molecular analysis was performed with RNA sequencing, bioinformatics, qRT-PCR, and Western blotting. The ultrastructure of mitochondria was examined using transmission electron microscopy.

**Results:**

We first successfully generated HMGB1 KO in mouse ESCs and showed that HMGB1 function is dispensable for both cell viability and pluripotency maintenance, while it is required for the cell proliferation boost during the naïve-to-primed pluripotency transition. Molecular and transcriptomic analysis identified the involvement of HMGB1 in the regulation of energy metabolism processes by regulating mitochondrial structure and function, as well as ROS homeostasis. Loss of HMGB1 function in mouse ESCs results in altered mitochondrial structure and excessive ROS production. HMGB1-dependent elevated ROS levels negatively regulate cell proliferation during the transition from naïve to primed pluripotency *in vitro*.

**Conclusion:**

While HMGB1 deficiency does not impair self-renewal in the naïve state, it causes a marked reduction in proliferation as cells advance to primed pluripotency. Our findings thus identify HMGB1 as a key regulator of mitochondrial integrity and ROS homeostasis during the naïve-to-primed pluripotency transition.

## 1 Introduction

The main characteristics of pluripotent stem cells, the capacities to self-renew and differentiate into all cell types of adult organism, are controlled by the core pluripotency transcription factors Oct4, Nanog, and Sox2 (Chen et al., 2008; Hall et al., 2009; Ng and Surani, 2011). During early embryonic development, pluripotent cells of the epiblast transit through different pluripotency states designated as naïve, formative, and primed. Mouse ESCs are derived from the epiblast of the preimplantation blastocyst and maintained in the naïve state of pluripotency. In culture, their maintenance depends on active leukemia inhibitory factor (LIF)/STAT3 and WNT (GSK3 inhibitor) signaling, as well as on MEK signaling inhibition (Evans and Kaufman, 1981; Qi et al., 2004; Varzideh et al., 2023; Weinberger et al., 2016). *In vitro*, ESCs can be differentiated through the formative epiblast-like stem cells (EpiLCs) into epiblast stem cells (EpiSCs) representing the state of primed pluripotency (Brons et al., 2007; Guo et al., 2009; Smith, 2017). EpiSCs exhibit more restricted differentiation potential, lacking the competency to contribute to chimeras following blastocyst injection (Brons et al., 2007; Kojima et al., 2014; Tesar et al., 2007). This transition from naïve to primed pluripotency is accompanied by a dramatic increase in proliferation rate and overall metabolic activity, as well as a significant remodeling of mitochondrial structure and function (Minchiotti et al., 2022; Romayor et al., 2022; Yousefi et al., 2019). Nevertheless, the molecular mechanisms that trigger and coordinate these extensive changes remain largely unknown.

HMGB1 is an HMG-box protein which plays important roles in various cellular processes, acting in the nucleus as a non-histone DNA-binding chromatin regulator to modulate gene expression, and in the cytoplasm and extracellular space–as a pro-inflammatory cytokine modulating immune responses (Chen et al., 2004; Chikhirzhina et al., 2020; Chikhirzhina et al., 2021; Raucci et al., 2019; Stros, 2010; Yanai et al., 2013; Yuan et al., 2024). To date, the function of HMGB1 in pluripotency, as well as underlying molecular mechanisms, remains to be elucidated. Previous studies have reported that HMGB1 is not required for self-renewal of human ESCs which are in the primed pluripotency state, having only a minor impact on their proliferation rate and cell cycle progression, while playing a role in neuroectodermal differentiation (Bagherpoor et al., 2017). Given that mouse and human ESCs represent distinct pluripotency states characterized by significant differences in cellular metabolism, mitochondria structure, and proliferation kinetics (Minchiotti et al., 2022; Romayor et al., 2022; Yousefi et al., 2019), the role of HMGB1 in naïve pluripotency and during the transition to the primed pluripotency state still requires clarification.

On the other hand, several studies have indicated that HMGB1 plays an important role in regulating mitochondrial structure and biogenesis in differentiated and cancer cell types (Tohme et al., 2017; Yang et al., 2020; Zhong et al., 2019). For instance, tumors lacking HMGB1 display markedly reduced mitochondrial biogenesis and severe mitochondrial dysfunction (Tang et al., 2011). The balance between mitochondrial fission and fusion is critically important for maintaining pluripotency in stem cells (Zhong et al., 2019). In pancreatic cancer cells, HMGB1 acts as a positive regulator of mitochondrial biogenesis (Yang et al., 2020). Furthermore, HMGB1 enhances mitochondrial function in mouse embryonic fibroblasts (MEFs) by directly regulating Hspb1, a component of the stress response system that counteracts mitochondrial dysfunction by promoting autophagy and mitochondrial fusion (Tang et al., 2011).

In this study we have explored the role of HMGB1 in regulating structure and function of mitochondria–organelles that are known to be drastically remodeled during the naïve-to-primed pluripotency state transition (Bahat et al., 2018; D'Aniello et al., 2019). Our results demonstrate that while HMGB1 functions are dispensable for the maintaining the naïve pluripotency state, it becomes essential for sustaining the burst of cell proliferation during the transition to the primed pluripotency state *in vitro*. We provide evidence that HMGB1 mediates this function by preserving mitochondrial integrity and regulating ROS homeostasis.

## 2 Materials and methods

### 2.1 Animal experiments

For the teratoma assay, 8–10 weeks old (n = 6) female NUDE mice *NU–A/A Tyrc/Tyrc Foxn1nu/Foxn1nu* (The lab animal nursery, Pushchino, Russian Federation) were used. The detailed experimental protocol is described in Teratoma analysis section, and the completed ARRIVE checklist is provided in the Supplementary Material. All procedures involving animals were conducted in strict accordance with the animal welfare legislation of the Russian Federation and were approved by the Institutional Ethical Committee (Project Title: « Study of the role of HMGB1 and HMGB2 non-histone chromosomal proteins in maintaining pluripotency of mouse embryonic stem cells», Approval No. 03/25, Date 22 April 2025). The study is reported in accordance with the ARRIVE guidelines 2.0.

### 2.2 Mouse ESC culture

All reagents used, unless otherwise specified, were purchased from Thermo Fisher Scientific, Waltham, MA, United States. Mouse ESCs (line E14Tg2A, BayGenomics) were cultured on cell-adhesive plastic plates coated with 0.1% gelatin (Sigma-Aldrich, St. Louis, MO, United States) in the serum-/LIF-containing (SL) medium comprising KnockOut DMEM (Thermo Fisher Scientific, United States), 15% fetal bovine serum (FBS, HyClone, GE Healthcare Life Sciences, Marlborough, MA, United States), 100 U/mL penicillin, 100 μg/mL streptomycin, 2 mM L-glutamine, non-essential amino acids, 50 µM beta-mercaptoethanol (Sigma-Aldrich, St. Louis, MO, United States), and human leukemia inhibitory factor (LIF, 1:5,000), which was produced and titered in house. To obtain ESCs in the ground naïve state of pluripotency for subsequent differentiation, they were cultured in serum-free N2B27-2i-L (2iL) medium, composed of 1:1 mixture Neurobasal and DMEM/F12 media supplemented with 1xN2 and 1xB27 components, 100 U/mL penicillin, 100 mg/mL streptomycin, 2 mM L-Glutamine, 50 µM beta-mercaptoethanol (Sigma-Aldrich, St. Louis, MO, United States), 3 µM CHIR-99021 (Axon, Groningen, Netherlands), 1 µM PD-0325901 (Axon Medchem, Groningen, the Netherlands), and LIF (1:5,000). All cells were maintained at 37 °C in a standard CO_2_ incubator.

### 2.3 CRISPR/Cas9-mediated gene knockout

For the CRISPR-Cas9-mediated knockout of the *Hmgb1* gene, the modified pX330-U6-Chimeric_BB-CBh-hSpCas9 plasmid (Addgene #42230) carrying EGFP selection marker was used. Guide RNAs (gRNAs) were designed using the Benchling platform (www.benchling.com) The following gRNA-encoding sequences were used: Scr 5′-GCA​CTA​CCA​GAG​CTA​ACT​CA-3′ and *Hmgb1*: 5′- TCA​GAG​AGG​TGG​AAG​GTA​AG-3’. The sequences were cloned at the BbsI restriction sites of the aforementioned plasmid. Plasmid transfection was performed using Lipofectamine Stem Reagent according to manufacturer’s protocol. 48 h after transfection, cells were sorted using the S3e Cell Sorter (Bio-Rad) with an EGFP filter to isolate transfected cells. The sorted cells were seeded at low density in the SL media and cultured for 8–10 days to allow colony formation. Single colonies were then picked and expanded. Knockout efficiency was confirmed by Immunofluorescence and Western Blot (WB) analyses.

### 2.4 Transient transfection

Transient transfusion experiments were performed in 12-well tissue culture plates using FuGene HD transfection reagent (Promega, United States) or Lipofectamine Stem Cell reagent. Cells were seeded at a density of 30,000 cells per well in the SL media. Next day, the medium was replaced with OptiMEM Reduced-Serum Medium and 2–3 h later, the transfection mixture composed of 1 μg plasmid, 4 μL FuGene HD (Promega, United States), and 200 μL OptiMEM, was added to each well. 12 h post-transfection, the medium was replaced with fresh SL medium. After additional 48 h, the cells were harvested and analyzed by immunocytochemistry, RT-PCR, FACS, and other methods.

### 2.5 Immunocytochemistry

Adherent ESCs were washed twice with PBS and fixed in with 4% paraformaldehyde (PFA, Sigma-Aldrich, St. Louis, MO, United States) for 10 min at room temperature (RT), followed by three washes with PBS (Amresco, cat. E404-200TABS), permeabilization with 0.1% Triton-X100 (AppliChem, Darmstadt, Germany) in PBS, and blocking with 3% bovine serum albumin (BSA, Capricorn, Germany) in PBS for 30 min at RT. Permeabilized cells were incubated overnight at 4 °C with primary antibodies diluted in PBS containing 1% BSA (see Supplementary Table S1 for antibody details). Next day, cells were washed five times with PBS and incubated with secondary fluorescently labeled secondary antibodies (see Supplementary Table S1) for 2 h at room temperature. After three additional washes with PBS, cells were counterstained with 4′,6-diamidino-2-phenylindole (DAPI; Sigma-Aldrich, United States) diluted in PBS (1:5,000) for 5 min at room temperature, and then DAPI solution was replaced with PBS containing 0.02% sodium azide. Immunofluorescence images were acquired using the EVOS™ FL Auto Imaging System equipped with DAPI, GFP, and RFP filter cubes. The negative controls were performed without the primary antibody incubation step.

### 2.6 Quantitative RT-PCR

Total RNA was isolated using TRIzol reagent according to the manufacturer’s protocol. For cDNA synthesis, 4–5 μg of total RNA was used. Complementary DNA was synthesized using MMLV reverse transcriptase kit (Evrogen, Russian Federation) following the manufacturer’s instructions. Quantitative RT-PCR (qRT-PCR) was performed using 5x qPCRmix-HS SYBR buffer (Evrogen, Russian Federation) on a CFX96 Touch Real-Time PCR Detection System (BioRad, United States). Each reaction was carried out in triplicate, and GAPDH was used as an endogenous control for normalization. The relative expression levels of target genes were calculated using the ΔCq method. dCq values were taken for statistical significance analysis using an unpaired two-tailed Student’s t-test, with p < 0.05 considered statistically significant.

### 2.7 Western blotting

Total cell lysates were prepared by washing cells twice with PBS and resuspending them in lysis buffer (50 mM Tris-HCl pH 6.8, 10% glycerol, l.1% SDS, 5% β-mercaptoethanol). Samples were heated at 95 °C for 5 min, and protein concentrations were quantified. 25–30 µg of total protein per well was separated by SDS-polyacrylamide gel electrophoresis in 13% polyacrylamide gel. Proteins were transferred to a nitrocellulose Hybond-ECL membrane (Thermo Fisher Scientific, Waltham, MA, United States) using semi-dry blotting at 20 V, 1 mA/cm^2^ for 180 min. The membrane was blocked with 5% dried milk, 0.1% Tween-20 (Helicon, Russian Federation) in PBS at room temperature for 60 min. Subsequently, the membrane was incubated overnight at 4 °C with primary-antibodies (see Supplementary Table S1) diluted in PBS containing 1% dried milk and 0.1% Tween-20. Next day, the membrane was washed 3 times for 10 min with PBS with 0.1% Tween-20) and incubated with secondary antibodies (see Supplementary Table S1) at room temperature for 2 h. Protein-antibody complexes were detected using SuperSignal Femto chemiluminescent substrate and the chemiluminescent signal was recorded using a ChemiDoc Touch Imaging System (Bio-Rad Laboratories). Gapdh was used as a loading control to ensure equal protein loading across samples. Analysis of protein quantity by Western blot was performed using the ImageJ.

### 2.8 Annexin V based apoptosis analysis

Cells were seeded at a density of 20,000 cells per well in a 24-well plate and cultured in the SL media for 48 h. Cells were stained with Annexin V-FITC Kit (Beckman Coulter, cat. IM3546, United States) and propidium iodide (PI, cat. P16063) according to the manufacturer’s protocol. Briefly, cells were harvested by trypsinization, washed, and resuspended in binding buffer (10 mM HEPES, 140 mM NaCl, 2.5 mM CaCl2, pH 7.4), followed by incubation with Annexin V-FITC and PI for 15 min at room temperature in the dark. Stained cells were analyzed by fluorescence-activated cell sorting (FACS) assay using the CytoFLEX flow cytometer (Beckman Coulter, Brea, CA, United States), and data were analyzed using CytoFLEX CytExpert software (version 2.3). Apoptotic cells were identified as Annexin V-positive, with early apoptotic cells being PI-negative and late apoptotic cells being PI-positive.

### 2.9 ROS detection

To assess the level of superoxide anion (SOA) in cells, dihydroethidium (DHE, Thermo Fisher Sci, Cat. D23107) and MitoSOX Red (MSR, Thermo Fisher Sci, Cat. M36008) reagents were used. Adherent ESCs were washed twice with PBS and incubated with either 5 nM DHE for 10 min or 2.5 µM MSR for 30 min at 37 °C in the dark. After incubation, cells were washed twice with PBS and analyzed using the EVOS® FL Auto fluorescence microscope (Thermo Fisher Scientific, United States). For FACS analysis, DHE- or MSR-stained cells were detached using 0.05% Trypsin/EDTA solution. After 5 min, trypsinization was stopped by adding two volumes of growth medium containing 10% FBS. Cell pellets were resuspended in PBS and analyzed using CytoFLEX flow cytometer (Beckman Coulter, Brea, CA, United States). Data were analyzed using CytoFLEX CytExpert software (version 2.3).

### 2.10 Quantitative analysis of active mitochondria in living cells

To assess mitochondrial content in living cells, Mitotraker-Red reagent (MTR, Thermo Fisher Sci, cat. M7512) was used. Adherent ESCs were washed twice with PBS and incubated with 50 nM MTR for 30 min in a CO_2_-incubator (5% CO_2_, 37 °C). After incubation, cells were washed twice with PBS, detached using standard trypsinization as described above, resuspended in PBS, and analyzed using a CytoFLEX flow cytometer (Beckman Coulter, Brea, CA, United States); data were analyzed using CytoFLEX CytExpert software (version 2.3).

### 2.11 TMRE mitochondrial membrane potential assay

To assess the mitochondrial membrane potential (MMP) in living cells, tetramethylrhodamine ethyl ester (TMRE, Abcam, cat. ab113852) was used. Adherent ESCs were washed twice with PBS and incubated with 100 nM TMRE for 30 min in a CO_2_-incubator (5% CO_2_, 37 °C). After incubation, cells were washed twice with PBS, harvested using standard trypsinization as described above, resuspended in PBS, and analyzed using a CytoFLEX flow cytometer (Beckman Coulter, Brea, CA, United States). As a positive control, cells were treated with 20 µM carbonyl cyanide 4-(trifluoromethoxy)phenylhydrazone (FCCP, Abcam PLC, Cat. 8206009), a mitochondrial oxidative phosphorylation uncoupler, to induce depolarization of the mitochondrial membrane. Data were analyzed using CytoFLEX CytExpert software (version 2.3).

### 2.12 Electron microscopy

For transmission electron microscopy (TEM), cells were fixed in 2.5% glutaraldehyde in 0.05 M cacodylate buffer (pH 7.3) containing 8.5% of sucrose. After primary fixation, cells were post-fixed in 2.0% OsO_4_ in the same buffer, dehydrated in an ascending series of ethanol, and embedded in a Spurr’s low-viscosity resin (Electron Microscopy Sciences, United States) according to the manufacturer’s recommendations. Ultrathin sections were cut using a Reichert Ultracut-E ultramicrotome (Austria), contrasted with uranyl acetate and lead citrate and examined using a Libra 120 electron microscope (Carl Zeiss, Germany) operating at 80 kV.

### 2.13 RNA extraction and transcriptome sequencing

Total RNA was isolated from the ESCs using Trizol reagent (Sigma-Aldrich, MA, United States) following the manufacturer’s instructions. The RNA was resuspended in DEPC-treated water and were assessed for concentration and quality by spectrophotometry and gel electrophoresis. RNA sequencing was performed using the BGISEQ-500 High-throughput Sequencing Platform (BGI, Beijing, China). Salmon protocol of quantifying transcript abundance from RNA-seq reads was used (Patro et al., 2017). Raw counts were processed and normalized using the following R Statistical Software (v4.3.2; R Core Team, 2023): tximport (https://github.com/thelovelab/tximport), genefilter (https://bioconductor.org/packages/genefilter), GenomicFeatures (https://github.com/Bioconductor/GenomicFeatures), and DESeq2 (https://github.com/thelovelab/DESeq2). Differential gene expression analysis was performed using DESeq2, and results were visualized using volcano plots generated with the SRplot resource (https://www.bioinformatics.com.cn/en?p=5). Gene ontology (GO) and Kyoto Encyclopedia of Genes and Genomes (KEGG) pathway enrichment analyses were conducted using SRplot resource to identify significantly enriched pathways among differentially expressed genes (DEGs).

### 2.14 Teratoma analysis

ESCs were washed with PBS (pH 7.5), harvested with 0.05% trypsin for 10 min at 37 °C, and collected by centrifugation at 1,200 *g* for 2 min. Cell were washed, resuspended in PBS, and injected subcutaneously (10^6^) into the hind limb of female NUDE mice. When tumor diameters reached approximately 1.5 cm (after 3–4 weeks), the mice were euthanized by cervical dislocation without anesthesia. Teratomas were then removed and fixed in 4% paraformaldehyde in PBS overnight at 4 °C. The detailed experimental protocol is provided in the ARRIVE checklist in Supplementary Material. Fixed teratomas were cut into 5 mm pieces, dehydrated through a graded ethanol series (70%, 80%, 96%), followed by isobutanol and two changes of xylene (1 h each at room temperature). Tissues were then incubated in 50% paraffin: 50% xylene for 1 h, followed by two changes of 100% paraffin (1 h each at 56 °C). Paraffin-embedded tissues were sectioned into 5 µm slices using a Leica RM2235 microtome (Leica Biosystems, Wetzlar, Germany). Sections were dried overnight at 37 °C on microscope slides, deparaffinized in xylene (twice for 5 min at room temperature each), and rehydrated through a graded ethanol series (96%, 80%, 70%) for 3 min each and distilled water for 1 min. For histological staining, slides were included in hematoxylin for 5 min, washed in excess water for 10 min, and stained in eosin for 5 min, followed by a final washing in distilled water for 1 min. Sections were dehydrated a graded ethanol series (70%, 80%, 96%) for 3 min, cleared in xylene (twice), and mounted into Canadian balsam under a coverslip. Slides were dried overnight at 37 °C. Teratoma histological sections were analyzed using the EVOS Cell Imaging Systems (Thermo Fisher Scientific, Waltham, MA, United States).

### 2.15 ESC-to-EpiSCs transition

ESCs were cultured for 1 week in the 2iL medium on a poly-l-ornithine (Sigma-Aldrich, United States)-coated plates. For transition into EpiSCs, ESCs were seeded on fibronectin-coated plates (10 μg/mL, Merck, Darmstadt, Germany) and cultured in the EpiSC-medium composed of a 1:1 mixture Neurobasal and DMEM/F12 media and supplemented with 1xN2 and 1xB27 components, activin A (20 ng/mL), bFGF (12 ng/mL), and 1% knockout serum replacement (KO-SR), as previously described (Hayashi et al., 2011). Transition was carried out for 10 days, and the medium was replaced every 2 days. On Day 3 of differentiation, the Wnt/β-catenin pathway inhibitor XAV 939 was added to the medium to a final concentration of 10 μM. On Day 6, cells were passaged using collagenase IV. On Day 8, the efficiency of transition was assessed by immunostaining on Oct6 and Oct4 markers.

### 2.16 Statistical analysis

Unless otherwise specified, data are presented as the mean of three replicates from one of three representative experiments. Results for qRT-PCR, cell counts, FACS are presented as mean ± standard deviation (SD). Statistical significance between the control and experimental groups was assessed using unpaired two-tailed Student’s t-test. A P-value < 0.05 was considered statistically significant.

Statistical parameters for the RNA-seq analysis, including log2-fold change, standard error (lfcSE), Wald statistic (stat), p-value, and FDR-adjusted p-value (padj) to control for false positives, are presented in Supplementary Table S2. The Benjamini–Hochberg FDR procedure was used to define the enrichment of each gene, with a corrected p-value (padj) < 0.05 considered statistically significant.

## 3 Results

### 3.1 HMGB1 is dispensable for pluripotency maintenance

**FIGURE 1:**
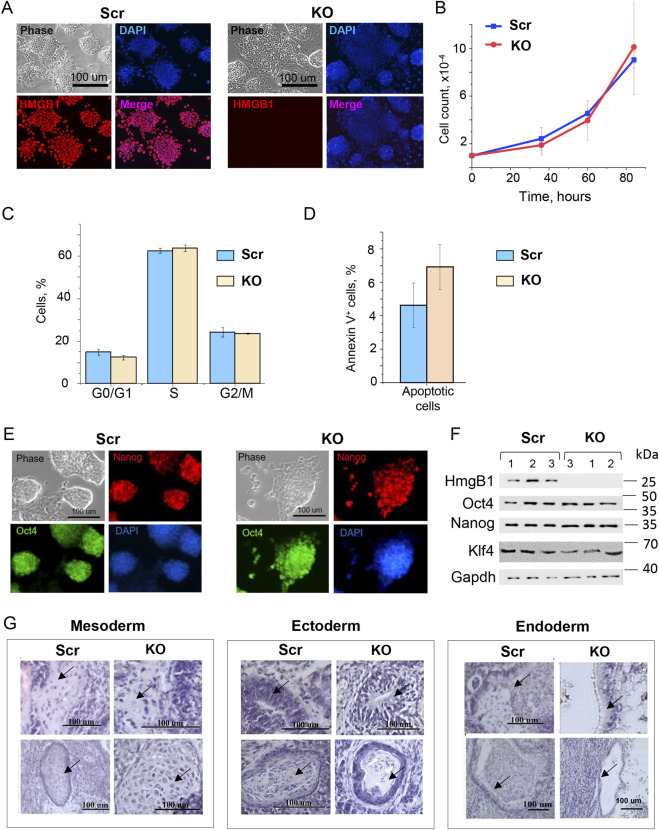
Loss of HMGB1 function does not affect mouse ESC self-renewal. **(A)** Immunocytochemistry affirming the loss of HMGB1 protein expression (red) in *Hmgb1*-knockout (KO) ESC compared with controls (Scr). Nuclei were counterstained with DAPI (blue). Cells were visualized with phase-contrast microscopy (phase). **(B)** Growth curves based on FACS analysis revealed similar proliferation rates of Scr and KO ESCs in serum-based media; values represented as means ± SD, n = 3 (biological replicates). **(C)** Similar cycle phase distribution of Scr and KO ESCs revealed by quantitative FACS analysis; values represented as mean ± SD, n = 3 (biological replicates). **(D)** Similar percentage of apoptotic and necrotic cells in Scr and KO ESCs, as determined by anti-Annexin V/propidium iodide staining and FACS analysis; values are represented as means ± SD, n = 3 (biological replicates). **(E)** Unaffected expression of Nanog (red) and Oct4 (green) proteins in KO ESCs, compared with Scr controls, revealed by immunocytochemistry. Nuclei were counterstained with DAPI (blue), and cells were visualized with phase-contrast microscopy (phase). **(F)** Loss of HMGB1 protein and unaffected levels of the pluripotency-associated Oct4, Nanog, and Klf4 in KO ESC clones confirmed by Western blot analysis. Samples of 3 Scr and 3 KO ESC clones were analyzed. Gapdh was used as a loading control, proteins molecular weight marker (kDa) is indicated on the right. **(G)** KO ESCs differentiate into cells of all three germ layers. Analysis of hematoxylin- and eosin-stained histological sections of Scr and KO ESC-derived teratomas identified chondroblasts and chondrocytes (mesoderm), neuroepithelial cells (ectoderm), and intestinal epithelium (endoderm) (indicated by arrows); scale bar – 100 µm.

To assess the role of HMGB1 in mouse ESC self-renewal and differentiation, we generated *Hmgb1*-knockout (KO) ESCs (hereafter referred to as KO ESCs), using CRISPR/Cas9 technique. In our study, guide RNAs were specifically designed to minimize potential off-target effects (Iyer et al., 2018; Smith et al., 2014). According to Benchling.com, none of the top 15 predicted off-target sites correspond to protein-coding genes or functional non-coding RNAs. Three independent KO clones and three scrambled (Scr) ESC clones were established. The KO ESCs remained viable and revealed no difference from Scr ESCs in proliferation rates when cultured either in the serum-based SL (Figures 1A,B) or in serum-free 2iL media (Supplementary Figure S1A). Furthermore, cell cycle phase distribution (Figure 1C; Supplementary Figure S1B) and percentage of apoptotic and necrotic cells were not statistically different in these genotypes (Figure 1D; Supplementary Figure S1C).

Next, we analyzed pluripotency status of HmgB1-deficient ESCs by evaluating the expression of pluripotency markers and performing teratoma formation assay. Immunocytochemistry and Western blot analysis revealed that the protein levels of key pluripotency markers Oct4, Nanog, and Klf4 were comparable in KO and Scr ESC clones (Figures 1E,F). However, a moderate downregulation of Oct4 and Klf4 mRNAs was observed in KO ESCs maintained in the SL medium, but not under the serum-free 2iL conditions (Supplementary Figures S1D,D’). Overall, the core pluripotency network appeared largely intact upon HmgB1 function loss. Teratoma assay confirmed that KO ESCs retain pluripotency, as the tumors contained tissue types belonging to all three germ layers: neuroepithelial cells (ectoderm), mesenchymal chondroblasts and chondrocytes (mesoderm), and intestinal epithelium (endoderm) (Figure 1G).

### 3.2 HMGB1 supports cell proliferation boost during the naïve-to-primed pluripotency transition

We next analyzed whether HMGB1 is involved in regulating the transition of ESCs toward primed pluripotency. A well-established model of the naïve-to-primed pluripotency transition, which mimics the epiblast state around implantation, is the *in vitro* conversion of ESCs into epiblast stem cells (EpiSCs) through an intermediate epiblast-like stem cells (EpiLCs) representing the formative pluripotency state (Hayashi et al., 2011; Smith, 2017; Tosolini and Jouneau, 2016). This transition is characterized by cell proliferation boost and changes in energy metabolism, particularly a shift from hybrid metabolism, involving both oxidative phosphorylation (OXPHOS) and glycolysis, to a predominantly glycolytic metabolism (Zhou et al., 2012).

**FIGURE 2:**
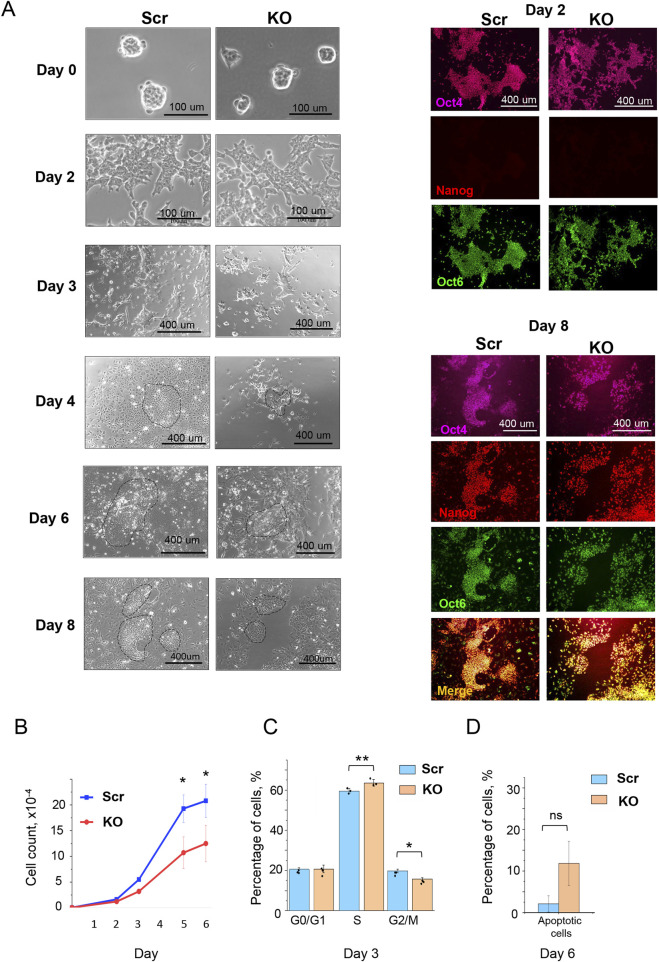
HMGB1 is required for proliferation during the naïve-to-primed pluripotency transition. **(A)** KO ESCs differentiated into EpiLCs (Day 2) and EpiSCs (Day 8) with the same efficiency as Scr ESCs, as determined by immunostaining for Oct4 (purple), Nanog (red), and Oct6 (green) markers. Phase-contrast microscopy images document progression of Scr and KO cell colonies from the ground state pluripotency (Day 0), through the formative EpiLC state (Day 2), to the primed EpiSC state (day 8). Colony boundaries are indicated by dashed lines **(B)** Cell proliferation curves indicating a significant decrease in proliferation of KO cells starting from Day 3 of differentiation toward EpiSCs, compared with the control Scr cells. Values are presented as mean ± SD, n = 3 (biological replicates), *p < 0.005. **(C)** Altered cell cycle phase distribution in KO cells on Day 3 of differentiation toward EpiSCs, as determined by FACS analysis. Values are presented as mean ± SD, n = 3 (biological replicates), *p < 0.006, **p < 0.05. **(D)** Percentage of apoptotic cells in KO cells on Day 6 of EpiSC differentiation compared with Scr cells, defined by anti-Annexin V antibody and propidium iodide staining, followed by FACS analysis. Values are means ± SD, n = 3 (biological replicates).

When cultured under the ground state 2iL conditions prior to the induction of differentiation to EpiSCs (Day 0), both KO and Scr ESCs formed spherical colonies and showed Oct4^+^/Nanog^+^/Oct6^–^ phenotype (Figure 2A; Supplementary Figure S2). By Day 2 of the transition, cells of both genotypes downregulated Nanog, acquired typical EpiLCs morphology (Figure 2A), and showed similar proliferation rates (Figure 2B) (Hamilton and Brickman, 2014; Kang et al., 2017; Molotkov et al., 2017). However, starting from Day 3 of the differentiation, the KO cell population displayed a reduced (down to 40%) proliferation rate which was monitored through 6-day passage-free culturing in parallel with Scr cells. Analysis of cell cycle phases distribution within the KO population revealed an increase in the S phase along with a reduction of cell number in the G2/M phase, compared to Scr cell population (Figure 2C; Supplementary Figure S3A). Consequently, from Day 4 to Day 6 of differentiation, the resulting KO EpiSC colonies appeared significantly smaller than Scr counterparts (Figure 2A), consistent with the observed differences in proliferation rates. On the other hand, neither a visible increase in cell death during the whole ESC-to-EpiSC transition process (Figure 2A), nor upregulation of the apoptosis marker Annexin V were revealed in KO cells on Day 6, the last day before passaging (Figure 2D). Despite the proliferation defect, KO cells were able to attain the primed pluripotency state (Figure 2A), as evidenced by the presence of Oct6^+^/Nanog^+^/Oct4^+^ cells on Day 8 (Figure 2A). Thus, our data suggest that while the differentiation into EpiSCs proceeds properly, proliferation during this transition is severely affected in KO cells.

### 3.3 HMGB1 involvement in regulation of energy metabolism processes

**FIGURE 3:**
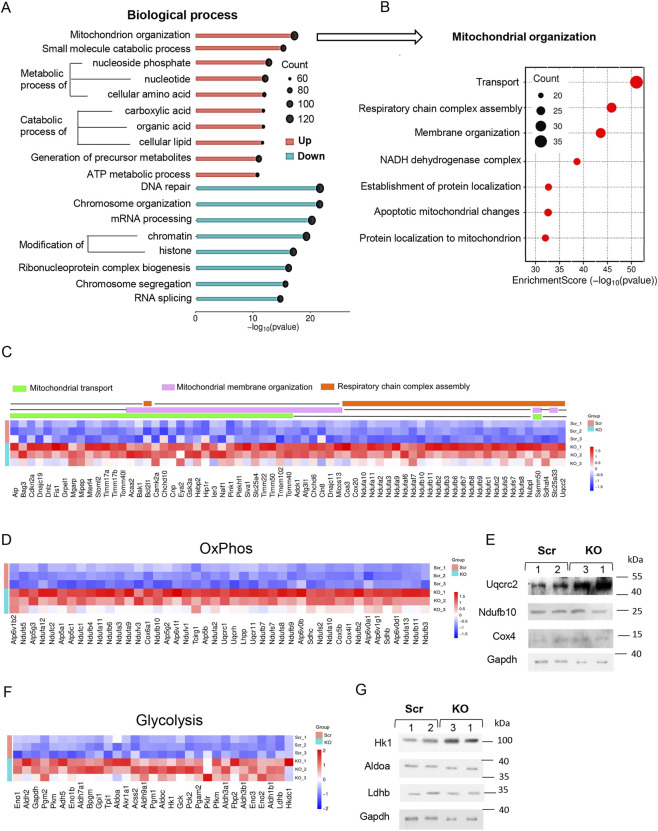
Transcriptome analysis reveals upregulated mitochondrial metabolism genes and downregulated chromatin-associated genes in KO ESCs. **(A)** Gene ontology (GO) enrichment analysis revealed the top 10 upregulated and top eight downregulated biological process (BP) affected in KO ESCs versus Scr ESCs. **(B)** Enrichment within the mitochondrial organization BP identified several GO clusters, demonstrating significant upregulation of genes involved in mitochondrial transport, mitochondrial respiratory chain assembly, and mitochondrial membrane organization in KO ESCs. **(C)** RNA-seq heatmaps illustrating differentially expressed genes (DEGs) involved in mitochondrial transport, mitochondrial membrane organization, and respiratory chain complex assembly in KO ESCs. The p-value and log2 fold change thresholds are provided in Supplementary Table S2. **(D)** RNA-seq heatmaps illustrating DEGs involved in oxidative phosphorylation (OXPHOS) in KO ESCs. The p-value and log2 fold change thresholds are provided in Supplementary Table S2. **(E)** Western blot analysis reveals elevated levels of Uqcrc2 protein, a subunit of Complex III of the ETC, but unchanged levels of Ndufb10 (Complex I) and Cox4 (Complex IV) in KO ESCs, compared to Scr controls. MW markers (kDa) are indicated on the right. **(F)** Heatmaps of 31 DEGs involved in glycolysis in KO ESCs, including Hk1, Eno1, Eno2, Eno3, Aldoa, and Aldoac. The p-value and log2 fold change thresholds are provided in Supplementary Table S2. **(G)** Western blot analysis shows elevated levels of Hk1 protein, but unaltered Aldoa and Ldhb levels in KO ESCs, compared to Scr controls. MW markers (kDa) are indicated on the right.

To gain an insight into the molecular changes associated with the loss of HMGB1 function in ESCs, we performed RNA-seq transcriptome analysis. Principal component analysis (PCA) revealed distinct transcriptomic profiles between the KO and control Scr ESCs, with each group represented by three independent clones showing expression correlation between biological replicates (Supplementary Figures S4A,B). Differential gene expression (DEG) analysis identified 1,548 upregulated and 1928 downregulated genes in KO versus Scr ESCs (Supplementary Figure S4C; Supplementary Table S2; Supplementary Table S3; Supplementary Table S4). Gene ontology (GO) enrichment analysis using over-representation analysis (ORA) revealed several GO groups which were predominantly affected in KO ESCs. Mitochondrial organization, small molecule catabolic processes, metabolic processes of nucleoside phosphates, nucleotides, and cellular amino acids were the most upregulated GO groups, while DNA repair, chromosome organization, mRNA processing, and chromatin modification represented the most downregulated GO groups (Figure 3A; Supplementary Figures S4D–F). The mitochondrial organization GO group, predominantly upregulated upon HMGB1 loss, featured genes related to mitochondrial transport, mitochondrial membrane organization, electron transport chain (ETC) complex assembly, apoptotic mitochondrial processes, and protein localization to mitochondrion (Figures 3B,C). In addition, the analysis revealed an increased expression of genes encoding ETC complexes I-V (CI-V), glycolysis, and tricarboxylic acid (TCA) cycle pathways in KO ESCs (Figure 3D; Supplementary Figures S5A,B).

Considering that most of the subunits of all five ETC complexes (Ndufa2, 3, 10–13, Ndufb2-4, 6–11, Ndufs2, 5, 7, 8, Ndufv1, 3, Ndufc1, 2, Uqcrc1, 2, 10, 11, Rb, q, Cox4il, 5b, 6a1, 20) were significantly upregulated in KO ESCs, we assessed protein levels of several representative ETC subunits, including Ndufb10 (CI), Uqcrc2 (CIII), and Cox4 (CIV). Indeed, we observed a significant (approximately 2.5-fold) increase in Uqcrc2 protein levels, while protein levels of Ndufb10 and Cox4 were unchanged in KO ESCs (Figure 3E), suggesting that at least CIII activity is upregulated. Functional consequence of the CIII upregulation for OXPHOS activity remains to be determined, however, according to a previous report (Chen et al., 2019), this activation may lead to increased ROS levels.

According to RNA-seq analysis, glycolysis-associated mRNAs, including hexokinase 1 (Hk1), aldolase A (Aldoa), enolase 2 and 3 (Eno2, Eno3), phosphoglycerate mutase (Pgam2), and 6-phosphofructokinases (Pfkm, Pfkl) were significantly upregulated in KO ESCs (Figure 3F), indicating a compensatory activation of this glycolytic pathway to meet the demand for ATP production. Elevated expression of several genes identified by RNA-seq, including glycolytic Hk1 and Eno3, was confirmed by RT-PCR (Supplementary Figure S5C). In addition, we evaluated protein levels of several glycolytic enzymes. Western blot analysis confirmed a significant increase of Hk1, but not of Ldhb or Aldoa protein levels, in KO ESCs (Figure 3G). This discrepancy between the mRNA and protein levels of Ldhb and Aldoa may be attributed to the complexity of gene expression regulation, suggesting that while HMGB1 may participate in the transcriptional regulation of these genes, their expression might simultaneously be regulated by post-transcriptional mechanisms (such as RNA interference, post-translational modifications, and proteasomal degradation), which warrants further study. Hk1 is a key glycolytic enzyme, catalyzing the phosphorylation of glucose to produce glucose-6-phosphate, allowing glucose trapping within the cell and to direct it toward energy production (Farooq et al., 2023). Thus, the level of Hk1 expression serves as an indicator of glycolytic activation. All these data suggest that HMGB1 plays a critical role in maintaining cellular metabolic homeostasis. This regulatory role of HMGB1 could have implications during different stages of naive-to-primed pluripotency transition.

### 3.4 HMGB1 is involved in regulation of mitochondrial structure and function, as well as ROS homeostasis

**FIGURE 4:**
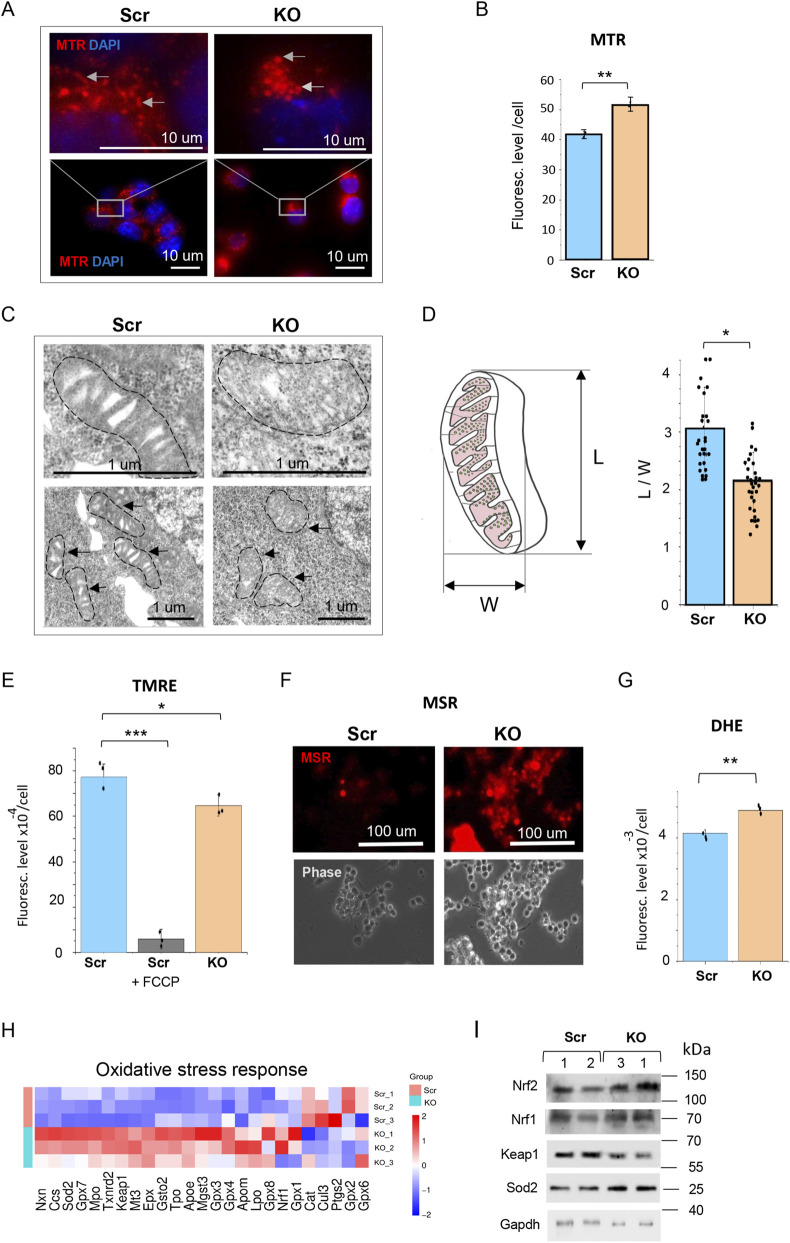
Abnormal mitochondrial structure and increased ROS generation in HMGB1-deficient ESCs. **(A)** Enlarged and round-shaped mitochondria in KO ESCs compared to Scr ESCs, detected by MTR-staining (red) in living cells and fluorescent microscopy. Nuclei were stained DAPI (blue). **(B)** The number of active mitochondria was significantly increased in KO ESCs, as demonstrated by MTR-staining and FACS analysis. Values are presented as mean ± SD, n = 3, **p < 0.01. **(C)** Swollen mitochondria with markedly reduced and disorganized cristae in KO ESCs compared to Scr control, revealed by transmission electron microscopy (TEM). Representative images are shown. The mitochondria are outlined with dashed lines. **(D)** Quantitative analysis of the mitochondrion length-to-width (L/W) ratio counted from TEM images. Values are presented as means ± SD, n = 3, *p < 0.001. **(E)** Mitochondrial membrane potential (MMP) was significantly reduced in KO ESCs, compared to control Scr ESCs, as revealed by TMRE staining and FACS analysis. The uncoupler FCCP was used as a positive control. Values are presented as mean ± SD, n = 3, *p < 0.02, ***p <0.001. **(F)** Elevated levels of mitochondrial superoxide (SOA/ROS) in KO ESCs compared to control Scr ESCs, demonstrated by MSR staining (red) in living cells, followed by fluorescent and phase-contrast microscopy (Phase). **(G)** The total cellular superoxide (SOA/ROS) was significantly elevated in KO ESCs compared to control Scr ESCs, as revealed by DHE and FACS analysis. Values are presented as mean ± SD, n = 3, **p < 0.01. **(H)** RNA-seq heatmaps showing DEGs associated with the cellular oxidative stress response in KO ESCs versus Scr controls. Expression of 18 genes, including Gpx3, Gpx4, Gpx7, Sod2 and Nxn, was upregulated. **(I)** Western blot analysis revealed elevated levels of Nrf2 and Sod2 proteins and decreased levels of Keap1 protein in KO ESCs compared to control Scr ESCs. MW markers (kDa) are shown on the right.

The above RNA-seq analysis showed upregulation of ETC complexes in KO ESCs, which prompted us to investigate parameters of mitochondrial function in these cells. Staining with the mitochondria specific MitoTraker-Red (MTR) dye revealed significant alterations in mitochondrial structure in KO ESCs. Mitochondria in KO ESCs appeared to be predominantly compact and round-shaped, compared to the more elongated mitochondria of control (Scr) ESCs (Figure 4A). An overall quantitative evaluation of MTR signal by FACS assay indicated a significant increase (around 19%) in the mass of active mitochondria in KO ESCs, compared to Scr ESCs (Figure 4B). Electron microscopy analysis further confirmed significantly altered morphology of the organelle in KO ESCs, characterized by disorganized cristae structure and a rounded, swollen morphology (Figures 4C,D). In addition, we observed a significantly decreased (about 15%) mitochondrial membrane potential (MMP) in KO ESCs versus Scr ESCs, as evaluated by TMRE assay (Figure 4E). Analysis of mitochondrial and total superoxide anion (SOA) levels was performed using fluorescence-based probes–MitoSox-Red (MSR) and dihydroethidium (DHE), correspondingly. The levels of mitochondrial and total SOA, representing a major form of reactive oxygen species (ROS), were significantly elevated in KO ESCs in comparison with control ESCs (Figures 4F,G). These results indicate that HMGB1 is required for proper organization and function of mitochondria network in ESCs, and that loss of this function leads to significant alterations in mitochondrial organization, resulted in an excessive ROS generation.

It is known that ROS are involved in regulation of various aspects of stem cell biology, including self-renewal and differentiation of pluripotent stem cells, as well as pluripotency induction (Chaudhari et al., 2014; Sart et al., 2015; Sinenko et al., 2021; Sinenko and Tomilin, 2023; Skvortsova et al., 2022). RNA-seq analysis revealed upregulation of several factors of cellular antioxidant and redox regulation in KO ESCs, such as Sod2, Gpx3, Gpx4, Gpx7, Nxn, and Ccs (Figure 4H). This observation is consistent with the idea that excessive levels of mitochondrial ROS induce oxidative stress in KO ESCs. Furthermore, protein expression analysis by Western blot revealed significant upregulation of Nrf2 and, to a lesser extent, of Nrf1 protein, as well as downregulation of the Nrf2 negative regulator Keap1 in KO ESCs (Figure 4I). Notably, despite a moderate increase in Keap1 mRNA levels in KO ESCs, a significant decrease in its protein expression was observed (Figure 4H). This suggests the involvement of post-translational regulatory mechanisms, such as the modification of sensor cysteine residues followed by autophagic or proteasomal degradation of Keap1 (Taguchi et al., 2012; Tkachev et al., 2011). Such a reduction in the level of this repressor protein confirms the activation of the canonical oxidative stress response pathway aimed at Nrf2 stabilization and its subsequent nuclear translocation. Overall, Keap1/Nrf2 pathway is activated in KO ESCs, thereby inducing the expression of antioxidant enzymes such as superoxide dismutases (SOD1, SOD2), catalase (Cat), and glutathione peroxidases (GPX1 - GPX4) (Figure 4H) (Flynn and Melov, 2013; Fuse and Kobayashi, 2017). Accordingly, we have found that mitochondrial SOD2 protein is upregulated in KO ESCs, compared to Scr ESCs (Figure 4I), indicating that this enzyme, in cooperation with GPX or Cat enzymes, may consequently detoxify ROS in mitochondria and cytoplasm (Flynn and Melov, 2013). In sum, our data strongly suggest HMGB1 function in controlling proper mitochondrial structure, which is critically required to maintain proper redox homeostasis in ESCs. While these significant alterations in mitochondria organization and redox homeostasis do not affect self-renewal of KO ESCs, they become critical during the highly energy-dependent process of transition of ESCs into primed EpiSCs, accompanied by a shift from hybrid to a predominantly glycolytic energy metabolism.

### 3.5 HMGB1-dependent ROS regulates cell proliferation during the transition from naïve to primed pluripotency *in vitro*

**FIGURE 5:**
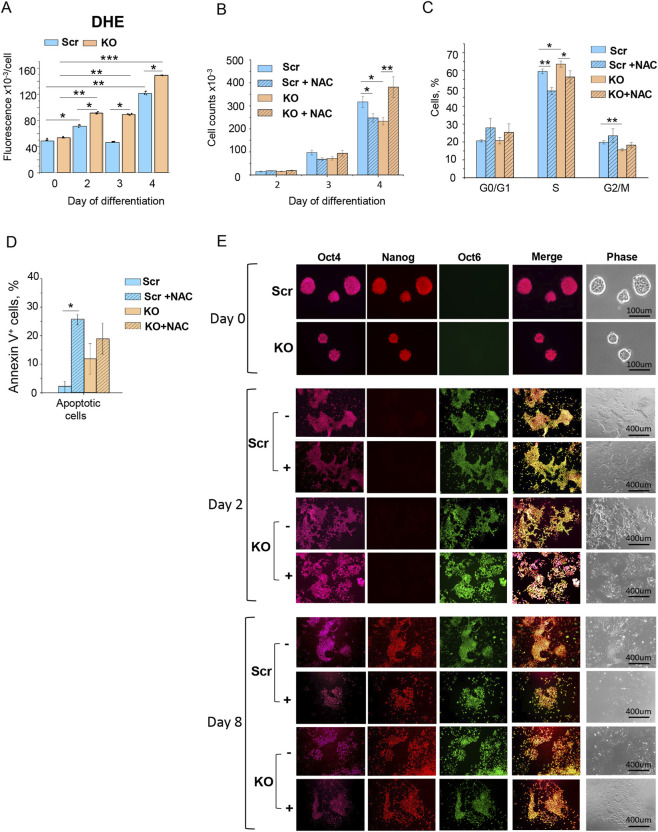
HMGB1 positively controls proliferation during the naïve-to-primed pluripotency transition via a ROS-dependent mechanism. **(A)** Increased levels of ROS in KO cells starting from day 2–4 of naïve-to-primed differentiation compared with control Scr cells, as revealed by DHE staining and FACS analysis. Data are presented as mean ± SD, n = 3 biological replicates, *p < 0.05, **p < 0.02, ***p < 0.007. **(B)** ROS scavenging by 2.5 mM NAC during naïve-to-primed differentiation restores proliferation rates of KO cells as determined by increased cell numbers on day 4 of the differentiation counted by FACS analysis. Data are presented as mean ± SD, n = 3 biological replicates, *p < 0.02, **p < 0.006. **(C)** ROS scavenging by NAC results in significant restoration of cell cycle S phase transition in KO cells at day 3 of differentiation to EpiSCs, but significantly reduces S phase in control Scr cells. Quantitative representation of FACS profiles of cell cycle phase distribution of the KO and Scr ESCs on Day 3 of differentiation upon NAC treatment. Values are presented as mean ± SD, n = 3 biological replicates *p < 0.05, **p < 0.01. **(D)** ROS scavenging does not alter apoptotic death rates in KO cells on Day 6 of differentiation to EpiSCs, but significantly increases the rate of apoptotic cells in control Scr cells. The percentage of apoptotic (PI^−^/AnV^+^) cells in KO vs. Scr cell populations on Day 6 of EpiSC differentiation upon NAC treatment, revealed by propidium iodide (PI) and Annexin V antibody staining, respectively, followed by FACS analysis. Values are presented as mean ± SD, n = 3 biological replicates, *p < 0.005. **(E)** ROS scavenging by 2.5 mM NAC during naïve-to-primed transition does not affect differentiation efficiency of KO cells compared to controls, as determined by properly expressing EpiLC- (Day 2) and EpiSC (Day 8) markers, Oct4^+^/Nanog^−^/Oct6^+^ and Oct^+^/Nanog^+^/Oct6^+^, respectively, as revealed by fluorescent and phase-contrast (phase) microscopy. Scale bar: 400 µm.

We sought that the increased levels of ROS resulted from ETC dysregulation is the reason for the decreased proliferation of KO cells during the ESCs-to-EpiSCs transition *in vitro*. First, we have assessed whether ROS levels dynamically change and are dependent on HMGB1 function during this transition. Since the proliferation defect could be observed starting from Day 3, we analyzed ROS levels during the first 4 days of the transition. According to DHE staining and FACS analysis, Scr control ESCs exhibit dynamic changes in ROS levels during the transition from the naive to the primed states (Figure 5A). This transition is characterized by specific stages in ROS generation in Scr cells: an initial increase by 1.5-fold (Days 0–2), a return to basal levels (Day 3), and a major elevation by 3.1-fold (from Day 4 onwards). In KO cells, ROS levels did not differ significantly on Day 0 but showed a marked increase from day 2 to day 4 of differentiation. Notably, the most substantial (2-fold) increase in ROS levels in KO cells compared to Scr controls was observed on Day 3, whereas on Days 2 and 4, the increase was approximately 1.3-fold (Figure 5A). The sustained elevation of ROS in KO cells on Days 3 and 4 (Figure 5A) correlates with reduced proliferation and altered cell cycle characteristics (Figures 2B–D).

To determine whether the ROS increase caused by the loss of HMGB1 contributes to the cell proliferation defect during the ESC-to-EpiSC transition, we scavenged ROS with 2.5 mM antioxidant N-acetylcysteine (NAC) treatment throughout the process. N-acetylcysteine (NAC) serves as a standard, widely used antioxidant, primarily by scavenging reactive oxygen species, such as hydrogen peroxide, and by reducing disulfide bonds in proteins and other sulfur-containing biomolecules (Aldini et al., 2018; Tyuryaeva and Lyublinskaya, 2023). We applied a low NAC concentration (2.5 mM) to avoid adverse effects (Ezerina et al., 2018).

Cells were counted from Day 2–4 of the transition using FACS analysis (Figure 5B). The treatment had no visible impact on cell numbers on Days 2 and 3, however, on Day 4, it essentially restored the number of KO cells to the levels of untreated Scr cells (Figure 5B). In addition, the distribution of cell cycle phases in NAC-treated KO cell population on Day 3 of the differentiation was also reverted to that of untreated Scr cells (Figure 5C; Supplementary Figure S6). Of note, the apoptotic cell death rate in the NAC-treated KO cell population was unchanged (Figure 5D). Importantly, the ESC-to-EpiSC transition of NAC-treated KO cells occurred normally, as evidenced by the presence of Oct6^+^/Nanog^+^/Oct4^+^ cells on Day 8 (Figure 5E). Taken together, these data strongly suggest that the increased ROS, mediated by the loss of HMGB1, is the primary cause of the observed proliferation defect during the naïve-to-primed transition.

Interestingly, ROS scavenging during the naïve-to-primed transition of Scr cells had the opposite effect, resulted in a reduced cell number on Day 4 (Figure 5B). This effect was likely due to increased apoptosis rates (Figure 5D) coupled with a decreased proliferation rate, as indicated by altered cell cycle progression and a decreased proportion of cells in S phase on the Day 3 (Figure 5C; Supplementary Figure S6). However, the ESC-to-EpiSC transition of Scr cells proceeded normally, as evidenced by the presence of Oct6^+^/Nanog^+^/Oct4^+^ cells on Day 8 (Figure 5E). This observation is consistent with the known requirement for tight regulation and optimal level of ROS as a signaling mediator in various cellular processes (Ivanova et al., 2021; Sies and Jones, 2020; Sinenko et al., 2021; Skvortsova et al., 2022; Ulfig and Jakob, 2024). It is likely that the precise, ROS-dependent regulation of proliferation occurs during the ESC-to-EpiSC transition, and that severely reduced ROS levels during this transition robustly impair cell proliferation.

Thus, our data indicate that optimal ROS levels are required for proper cell proliferation during the mid-stage of the ESC-to-EpiSC transition. The function of HMGB1 is essential to maintain mitochondrial ROS levels within this optimal range during this differentiation process.

## 4 Discussion

Our study revealed that HMGB1 function is not required for pluripotency maintenance of naïve-state mouse ESCs. Similarly, previous finding demonstrated lack of HMGB1 function in human primed-type ESCs (Bagherpoor et al., 2017). However, our data revealed an important function of HMGB1 in the positive regulation of cell proliferation during energy dependent process of transition between the naïve and primed states of pluripotency.

For the first time, we have identified that HMGB1 is required for proper structural organization and function of mitochondria of mouse ESCs. Loss of HMGB1 function leads to mitochondrial swelling, disorganization of cristae, and some increase in the mass of active mitochondrial network per cell. These changes are associated with a mild decrease in mitochondrial membrane potential and a significant increase in mitochondrial ROS levels. RNA-seq data reveal that, in addition to the expected downregulation of genes involved in chromatin associated processes, HMGB1 loss of function results in the activation of energy metabolism genes, including those involved in OXPHOS, glycolysis, mitochondrial organization, as well as genes related to nucleotide and amino acid catabolic processes.

Specifically, RNA-seq data showed that the expression of numerous genes encoding subunits of all ETC complexes was elevated in HMGB1-deficient ESCs, which may contribute to enhanced respiration and oxidative phosphorylation (Hock and Kralli, 2009; Scarpulla, 2008). However, we observed a significant increase in the protein level of Uqcrc2 (CIII), but not in Ndufb10 (CI) or Cox4 (CIV). This increase in the Uqcrc2 subunit expression may suggest an alteration, and possibly an enhancement, in the CIII functional activity. This is consistent with the established fact that CI and CIII are the primary sources of mitochondrial ROS (Andreyev et al., 2005; Brand, 2010). The elevated Uqcrc2 level could potentially facilitate a CIII state characterized by excessive electron leakage at the Qo site, thereby generating superoxide anions. This assumption, as well as the status of other ETC complexes and overall OXPHOS activation in this context, warrants further investigation.

HMGB1 is an architectural chromatin protein whose absence likely leads to altered chromatin accessibility at specific loci, thereby permitting transcriptional activation that is normally suppressed (Celona et al., 2011; Saunders et al., 2024; Starkova et al., 2023; Stros, 2010). Moreover, it has been reported that HMGB1 directly interacts with regions of mitochondrial DNA, including those encoding ETC components (Ito et al., 2015; Yang et al., 2020). Our studies demonstrate that the loss of HMGB1 leads to a consistent and coordinated upregulation of a mitochondrial and metabolic gene signature, suggesting that it is essential for maintaining the physiological repression of these metabolic programs in ESCs. The upregulation of ETC gene transcription, while not corroborated by the modest MMP decrease, suggests a possible compensatory mechanism in response to mitochondrial dysfunction. In accordance with previous studies, we propose that HMGB1 deficiency disrupts mitochondrial architecture, manifested as altered cristae organization and compromised membrane integrity (Tang et al., 2010; Wahid and Cunningham, 2025), thereby leading to a reduction in MMP. In this scenario, the cell may engage the corresponding mitochondrial biogenesis program in a compensatory effort to restore energy balance and maintain adequate energy metabolism, as well as the viability of HMGB1-deficient ESCs and their differentiated progeny.

RNA-seq data also reveal activation of several glycolytic genes in HMGB1-deficient ESCs, with a significant upregulation of the key enzyme hexokinase 1 at the protein levels. This may suggest that a significant dysregulation of energy metabolism and oxidative stress adaptation in these cells activates some compensatory and stress defense mechanisms (Robey and Hay, 2006). This metabolic phenotype resembles the significantly affected energy metabolism in HMGB1-deficient mice, which exhibit reduced body size at birth, multiple organ dysfunctions, and hypoglycemia, leading to early death (Calogero et al., 1999). Taken together, our data suggest that HMGB1 acts as a suppressor in the regulation of mitochondrial metabolic genes, and that the mechanism of this regulation warrant further investigation.

In agreement with increased ROS levels, we found a significant upregulation of Keap1/Nrf2 antioxidant response pathway and its target genes, Sod2 and Gpx enzymes, which neutralize the superoxide anion radical and hydrogen peroxide, respectively. This suggests that antioxidant response activation in HMGB1*-*dificient cells protects them from excessive ROS, thereby preserving their pluripotency. Importantly, depletion of HMGB1 in differentiated cells, such as MEFs, causes mitochondria abnormalities (Tang et al., 2011), similar to those observed in our study, including increased mitochondrial mass and decreased MMP. However, unlike MEFs, the loss of HMGB1 function in ESCs induces oxidative stress response but fails to activate the heat-shock-type stress response, which is typically characterized by upregulated expression of cytoprotective heat shock protein beta-1 (Hspb1) and its downstream targets, including mitophagy genes Pink1 and Vdac1 (Tang et al., 2011). These genes were not induced in HMGB1*-*deficient ESCs (Supplementary Figure S7). These findings suggest distinct roles for HMGB1 in MEFs and ESCs, specifically pointing to its role in ESCs in regulating mtROS levels.

Elevated ROS levels in HMGB1-deficient ESCs do not affect their proliferation rate, self-renewal, or differentiation potential. Given the absence of viability-affecting phenotypic abnormalities in ESCs our data suggest that increased ROS levels, resulted from impaired mitochondrial function following HMGB1 inactivation, modulate signaling events through oxidative eustress. That causes the plausible modification of signaling proteins rather than oxidative distress that would trigger severe macromolecular damage. Importantly, we identified that cell proliferation during naïve-to-primed pluripotency transition is controlled by ROS signaling. Through its role in maintaining mitochondrial integrity, HMGB1 helps maintain optimal ROS levels in different cell contexts. In particular, the ROS balance becomes essential for proliferation during the naïve-to-primed state transition, and HMGB1 inactivation-mediated ROS increase caused a suppression of proliferation, while overall the differentiation occurred properly. However, markedly reduced ROS levels during the naïve-to-primed transition of wild type cells also significantly suppress cell numbers due to increased levels of apoptosis and affected cell cycle progression, which sufficiently impairs differentiation. This suggests that optimal, tightly regulated ROS levels are essential for a proper naïve-to-primed pluripotency transition.

Mitochondria play key roles in energy metabolism, redox balance, ROS signaling, calcium homeostasis, apoptosis, and other cellular processes (Monzel et al., 2023; Spinelli and Haigis, 2018), and are critical for regulating cell fate decisions during embryogenesis, including the induction, maintenance, and differentiation of pluripotent stem cells (Arribat et al., 2019; Jasra et al., 2023; Lonergan et al., 2007; Sinenko and Tomilin, 2023). Mitochondrial functions and dynamics, such as OXPHOS, biogenesis, fission/fusion, and mitophagy processes are important for regulating PSC pluripotency quality, maintenance, and differentiation through retrograde signals (Lonergan et al., 2007; Mandal et al., 2011; Ren et al., 2020; Schmitz et al., 2025; Zhong et al., 2019). It has been shown that mitochondrial biogenesis plays a critical role during the differentiation of hESCs into endodermal lineages (Li et al., 2020; Lv et al., 2022). Mitochondrial ROS, known to act as redox signaling messengers, are involved in the regulation of various cellular and developmental processes, including those in stem cells (Ivanova et al., 2021; Sinenko et al., 2021; Tothova et al., 2007; Tsogtbaatar et al., 2020). Collectively, our study highlights the critical role of HMGB1 in regulating mitochondria structure, function, and, consequently, ROS generation in naïve-type mouse pluripotent stem cells, and reveal that HMGB1 is essential for maintaining proper mtROS levels, which are required to sustain cell proliferation rate during the transition to the primed pluripotency state.

## Data Availability

The datasets presented in this study can be found in online repositories. The names of the repository/repositories and accession number(s) can be found in the article/Supplementary Material.
