# Supplementary material for: HMGB1 regulates mitochondrial structure and reactive oxygen species balance during the transition from naïve to primed pluripotency
Source: Front Cell Dev Biol. 2026 May 12;14:1807454. doi: 10.3389/fcell.2026.1807454 (PMC13201526; doi:10.3389/fcell.2026.1807454)
Supplement: Supplementary file 1 [file DataSheet1.zip › Data Sheet 1.PDF]

ARRIVE 2.0 checklist

Study Design: 8-10 weeks old (n=6) female NUDE mice NU–A/A Tyrc/Tyrc Foxn1nu/Foxn1nu (The lab animal nursery, Pushchino, Russian Federation) were used. Each mouse received bilateral subcutaneous injections of viable cell suspensions in sterile PBS: 1×10^6 Scr control ESCs into the left hind limb and 1×10^6 HMGB1-KO ESCs into the right hind limb. This resulted in a total of 12 injections (6 per genotype). After 3–4 weeks, when any tumor in the cohort reached approximately 1.5 cm in diameter, mice were euthanized by rapid cervical vertebrae dislocation (without anesthesia) performed by a trained technician, and teratomas were carefully excised using aseptic surgical technique (scalpel and scissors), fixed, and processed for analysis.

Sample Size: A total of 6 athymic nude mice were used. Each mouse received bilateral subcutaneous injections: control Scr mESCs in one hind leg and experimental *HMGB1*^{KO} mESCs into the contralateral hind leg. This generated 6 biologically paired samples (n=6 per group). The sample size was chosen based on common practice in teratoma formation assay to account for biological variation and to be amenable to paired statistical comparison.

Inclusion and exclusion criteria: a) All generated teratomas were included in the analysis. The sole pre-established (*a priori*) inclusion criterion was successful teratoma formation, defined as the presence of a palpable and harvestable tumor mass at the endpoint. b) No animals or samples were excluded from the analysis. c) The final sample size was n = 6 biologically paired observations per genotype (6 control Scr-ESC teratomas and 6 HMGB1-KO ESC teratomas, each derived from one of the six mice used in the paired experimental design).

Randomisation: Randomisation was not used for group allocation. Animals were allocated to the control and experimental groups based on litter origin and similar body weight (±2 g) to minimize baseline variability. Importantly, to avoid cage-location bias, mice from both groups were co-housed in the same cage.

Blinding: The experimental group allocation was known only to the lead investigator during the setup phase. All subsequent procedures including cell injection, teratoma harvesting, and histological processing and scoring were performed by a separate technician who was blinded to the group identity. The blinding was lifted only after the statistical evaluation was completed by the lead investigator.

Outcome Measures: a) The study assessed: (1) successful teratoma formation rate, (2) gross morphology of excised teratomas, and (3) detailed histological analysis to confirm pluripotent differentiation into all three germ layers (ectoderm, mesoderm, and endoderm). Animal health and tumor burden were monitored daily, using digital calipers (length x width^2/2). Mice survival was controlled daily. Mice were euthanized at a predefined humane endpoint; therefore, survival was not an independent variable. b) This was an exploratory study and no formal hypothesis was tested.

Statistical Methods: The presence of each of the three germ layers (ectoderm, mesoderm, and endoderm) in a teratoma was confirmed if at least three distinct representative tissue structures were identified per histological section. This scoring generated categorical data. Histological analysis confirmed pluripotency by identifying derivatives of all three germ layers in 100% of teratomas from both groups; therefore, this parameter was not subjected to statistical testing.

Experimental Animals: Six athymic BALB/c nude female mice (*Crl:NU(NCr)-Foxn1*^{nu}; Mus musculus), aged 6–8 weeks and weighing 18–22 g, were used in the study. Animals were obtained from Jackson Laboratory (Bar Harbor, ME, USA). b) Mice were housed under specific pathogen-free (SPF) conditions and they were not genetically modified in this study. No animals had undergone any prior procedures; all were treatment-naïve before the experimental cell injections.

Experimental procedures. Six (n=6) female athymic Nude mice (8-10 weeks old) received bilateral subcutaneous injections of 1 x 10^6 Scr control ESCs (in PBS) into the right hind limb and 1 x 10^6 *HMGB1*-knockout ESCs (in PBS) into the hind limb. Three independent KO clones and three scrambled (Scr) ESC clones were used. Scr and KO ESCs were randomly assigned to mice in a paired design, so that each mouse received one unique Scr-KO ESC clone pair. Each clone was used in two different mice, resulting in biological duplicates. Thus, 6 Scr-derived and 6 KO-derived teratomas were obtained. When the largest tumor in the cohort reached approximately 1.5 cm (after 3–4 weeks), mice were euthanized by rapid cervical dislocation and teratomas were excised. Teratomas were fixed in 4% paraformaldehyde overnight at 4 °C, sectioned into ~5 mm pieces, and dehydrated through a graded ethanol series (70%, 80%, 96%), followed by clearing in isobutanol and two changes of xylene (1 hr each at room temperature). Tissues were then incubated in 1:1 mixture of paraffin: 50% xylene for 1 hr, followed by two changes of 100% paraffin (1 h each at 56°C). Paraffin-embedded tissues were sectioned into 5-μm slices using a Leica RM2235 microtome (Leica Biosystems, Wetzlar, Germany). Sections were dried overnight at 37°C, deparaffinized in xylene (twice for 5 min), rehydrated through a descending ethanol series (96%, 80%, 70%, 3 min each) to distilled water (1 min), and stained with hematoxylin for 5 min. After 10-min wash in running tap water, sections were counterstained with eosin for 5 min, briefly rinsed in distilled water, dehydrated through an ascending ethanol series (70%, 80%, 96%; 3 min each), cleared in xylene (twice for 5 min), and mounted with coverslips using Canadian balsam. Slides were dried overnight at 37°C. Histological analysis was performed using an EVOS Cell Imaging Systems (Thermo Fisher Scientific, Waltham, MA, USA)

d) Rationale. Subcutaneous injection in the hind limb is a standard site for teratoma assays. It facilitates easy, non-invasive monitoring of tumor growth, provides a well-vascularized environment for graft survival, and ensures clear anatomical separation from internal organs. The 3–4-week duration is based on established protocols and is sufficient for mESC-derived teratomas to reach an analyzable size and undergo differentiation into tissues of all three germ layers. The

use of immunodeficient athymic nude mice, which lack functional T-cells, prevents immune rejection of the murine ESCs, thereby enabling successful engraftment and teratoma development.

Results: All teratomas derived from both control (Scr, n=6) and experimental ($HMGB1^{KO}$, n=6) mESC lines successfully contained differentiated tissues representing all three germ layers (ectoderm, mesoderm, and endoderm), confirming pluripotent differentiation in 100% of samples. Therefore, no statistical comparison of this categorical outcome was required or performed.
