# Supplementary material for: HMGB1 regulates mitochondrial structure and reactive oxygen species balance during the transition from naïve to primed pluripotency
Source: Front Cell Dev Biol. 2026 May 12;14:1807454. doi: 10.3389/fcell.2026.1807454 (PMC13201526; doi:10.3389/fcell.2026.1807454)

For Fig 1F

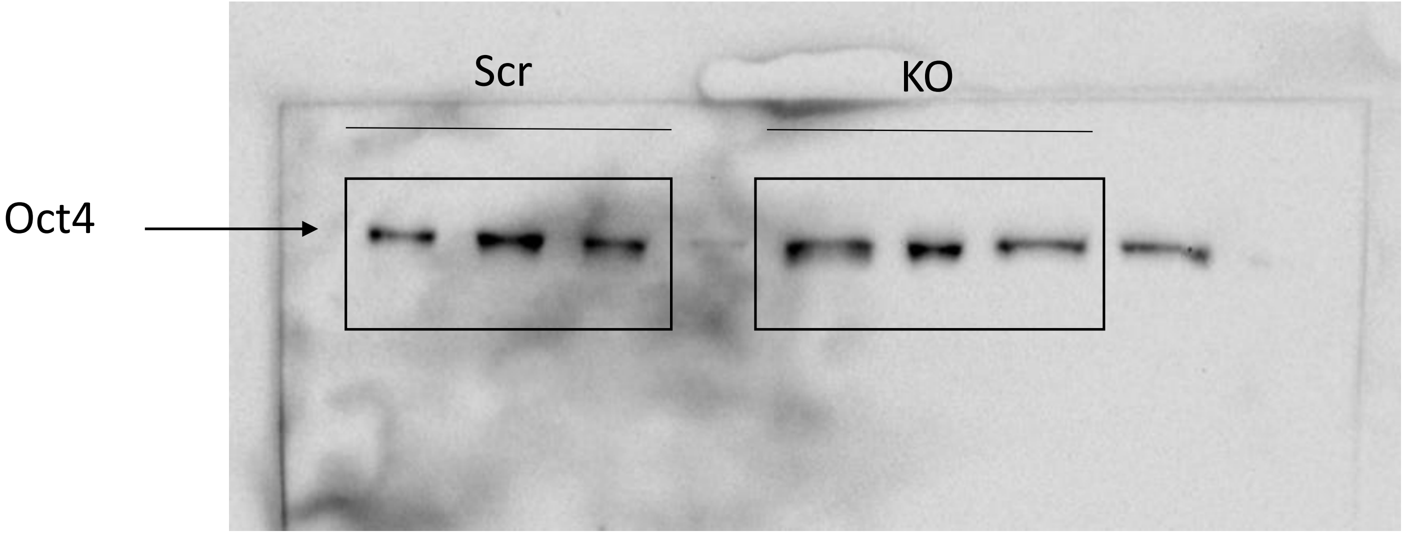

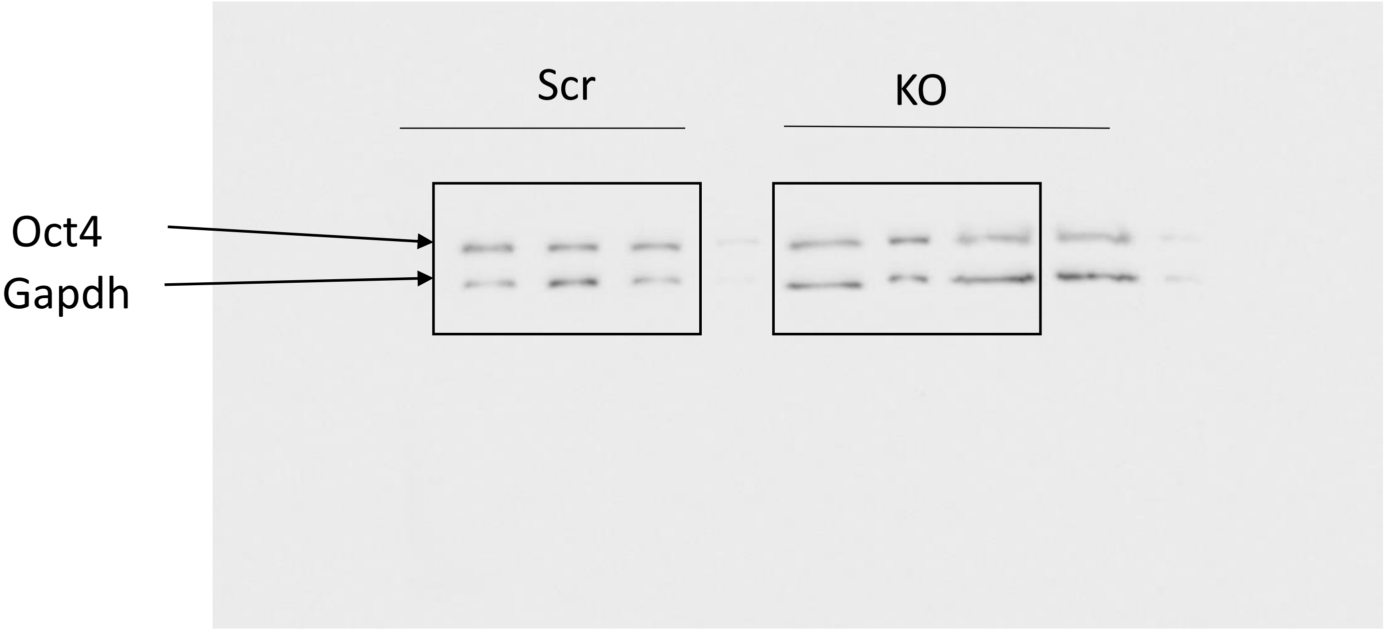

For Fig 1F

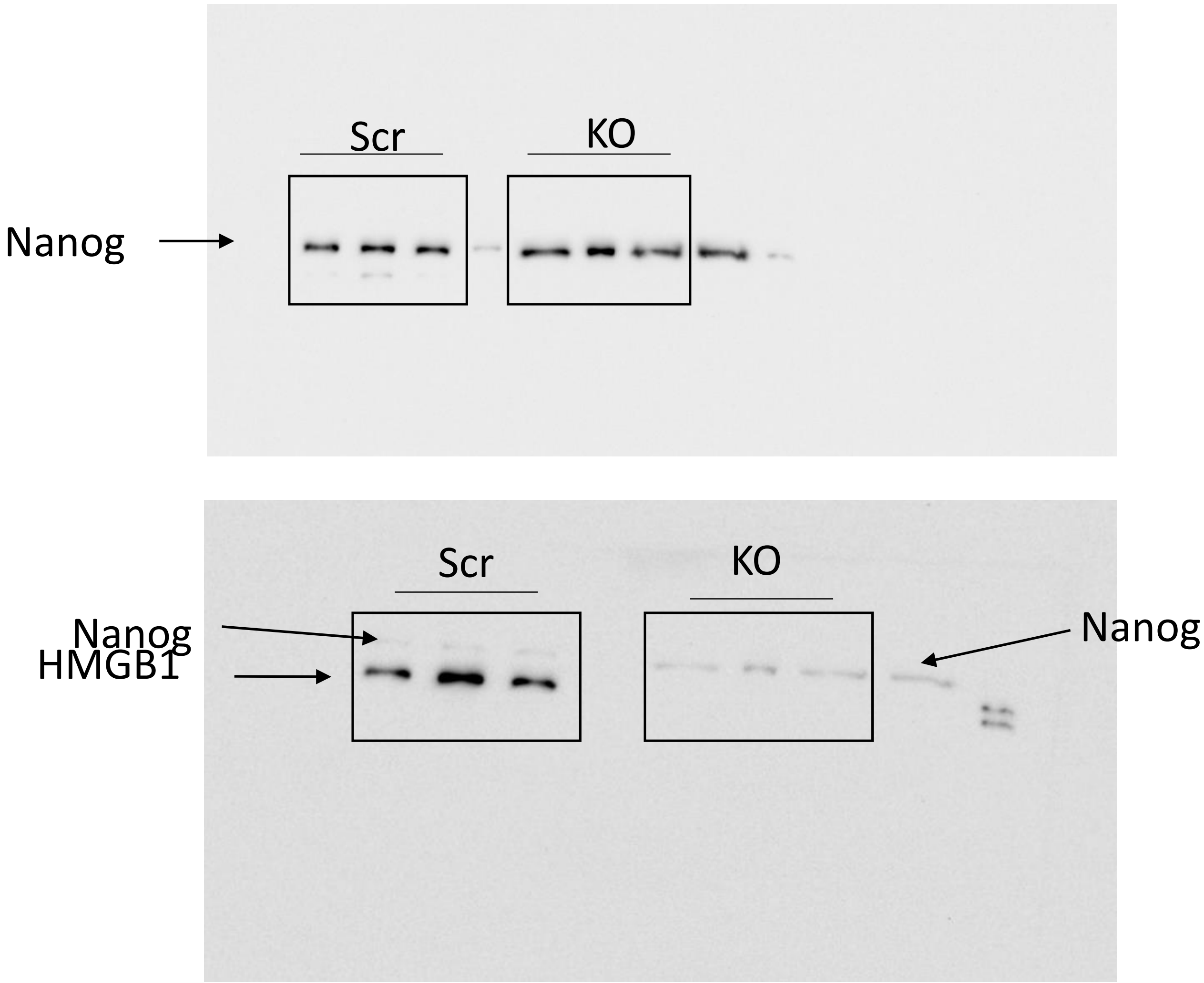

For Fig 1F

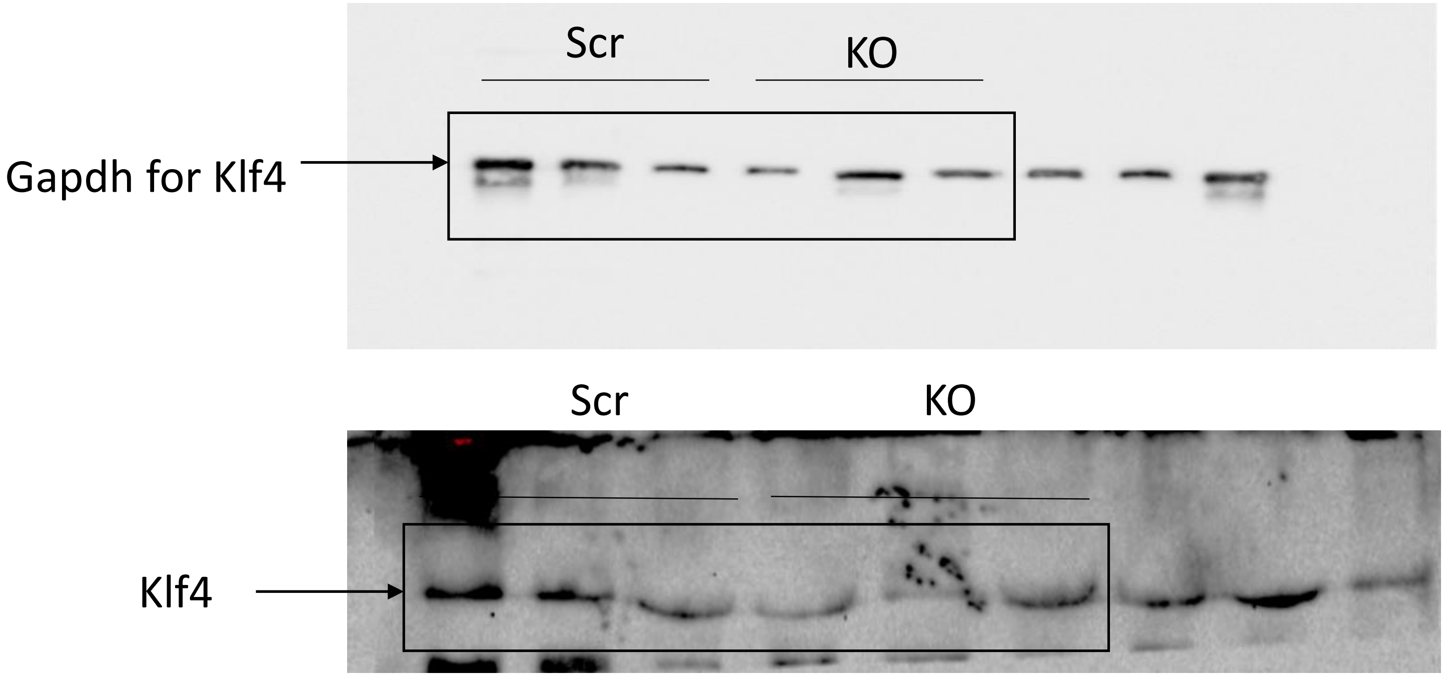

For Fig 3E

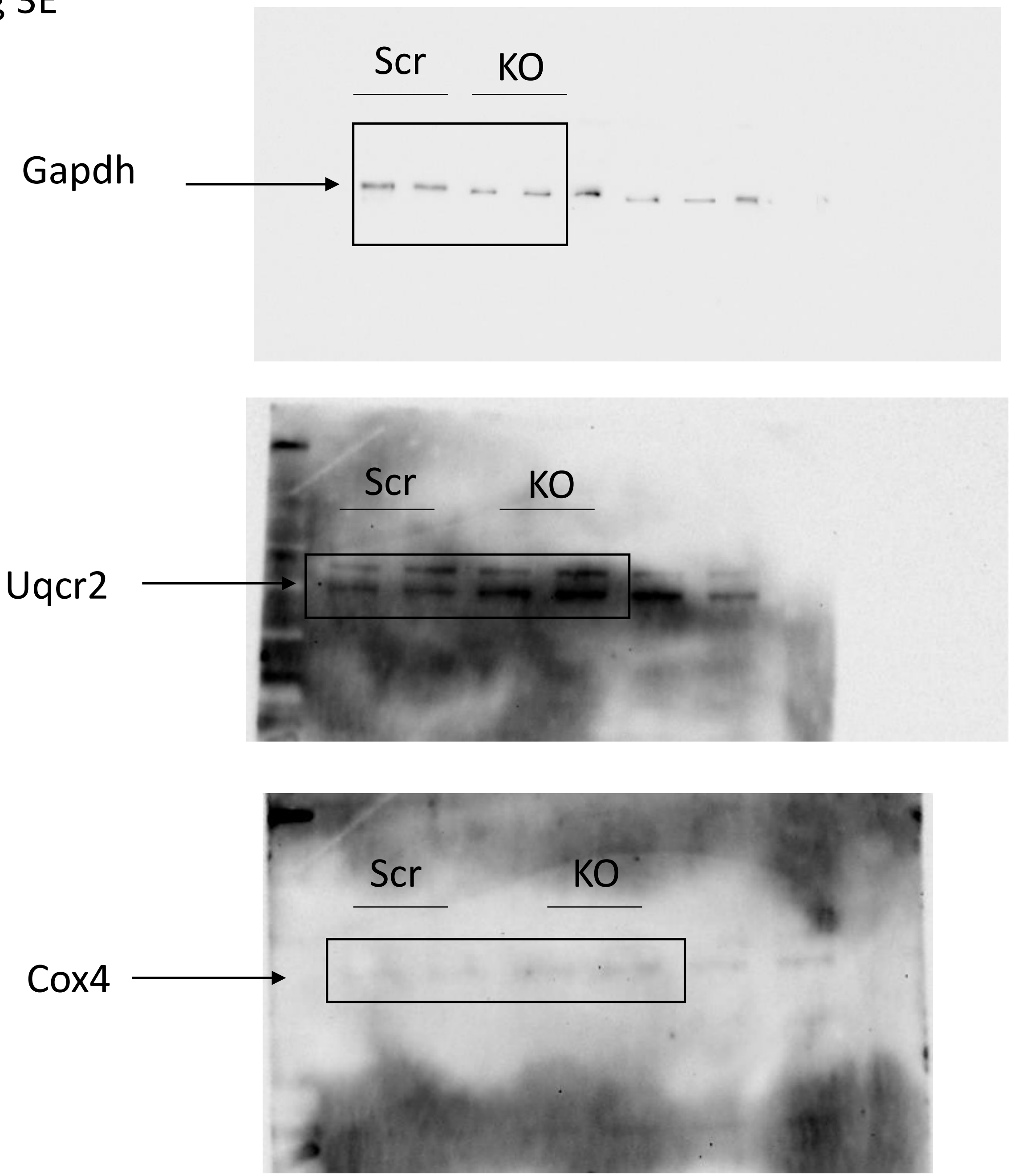

For Fig 3E

Gapdh for Ndufb10

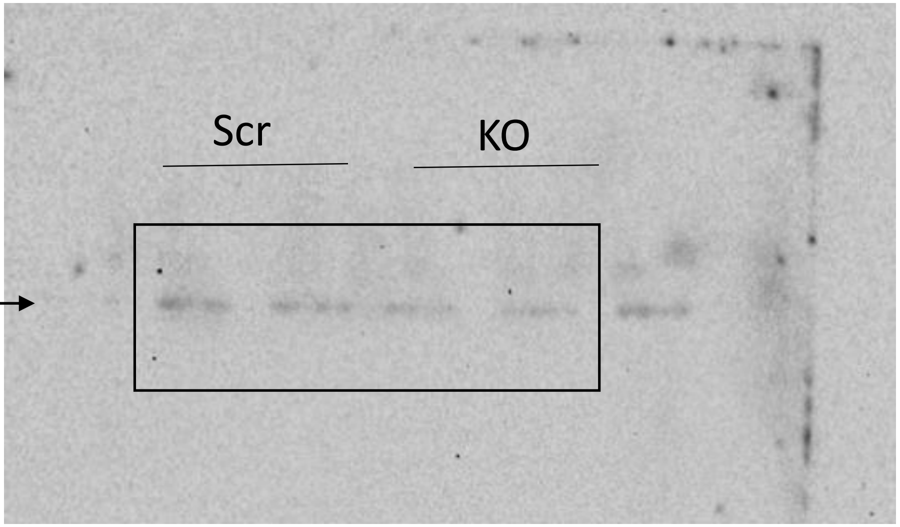

Ndufb10

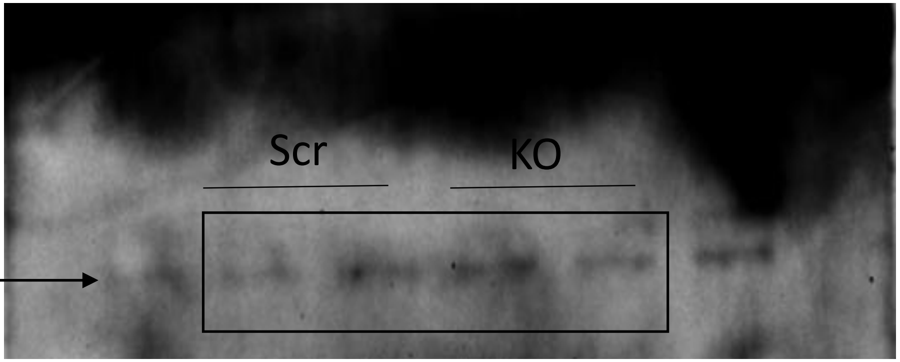

For Fig 3G

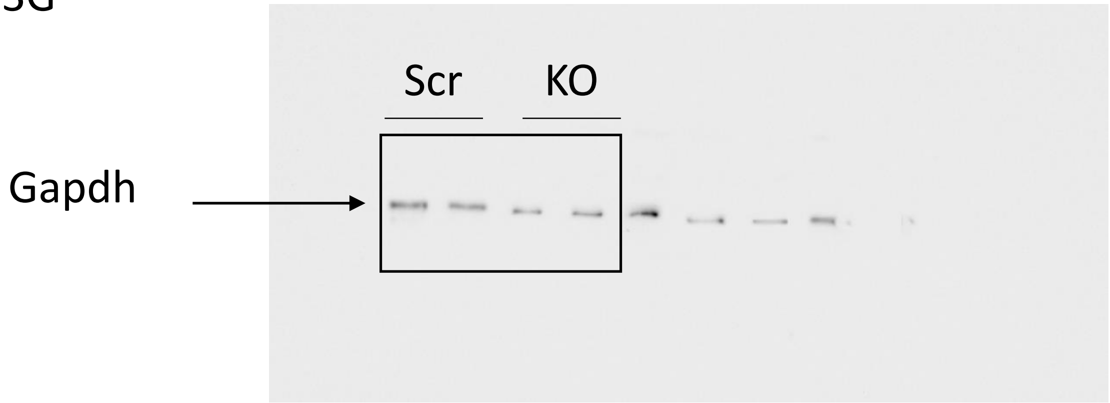

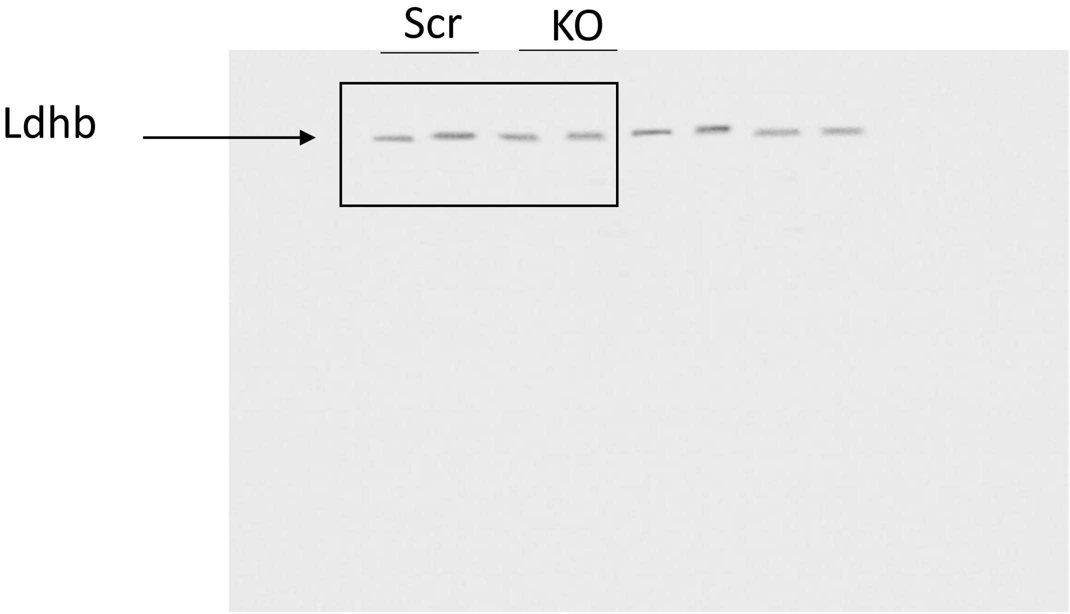

For Fig 3G

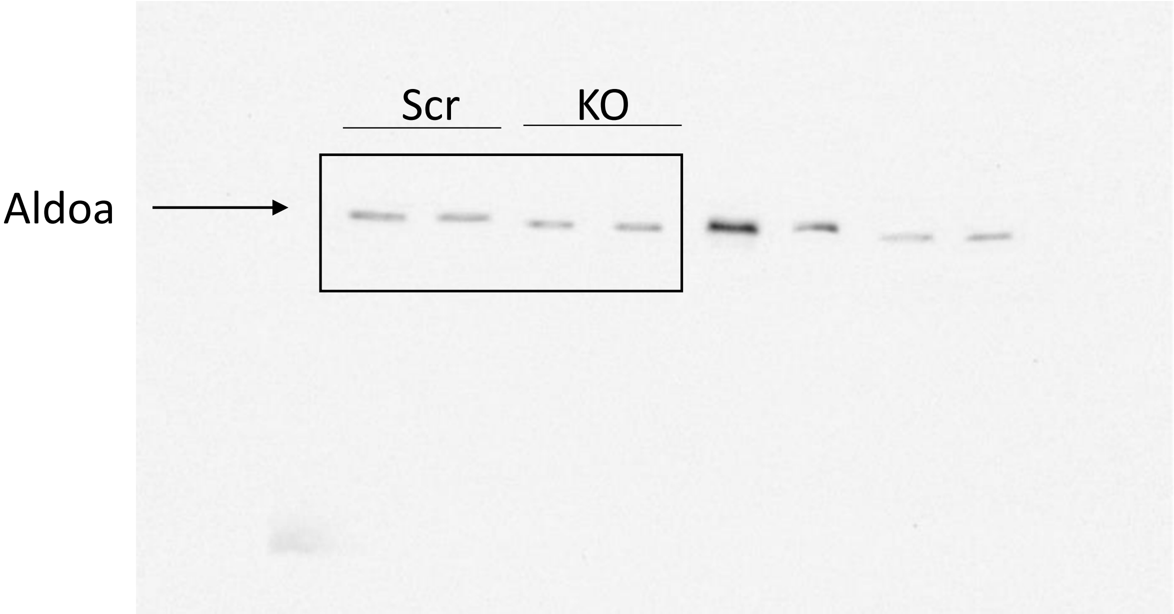

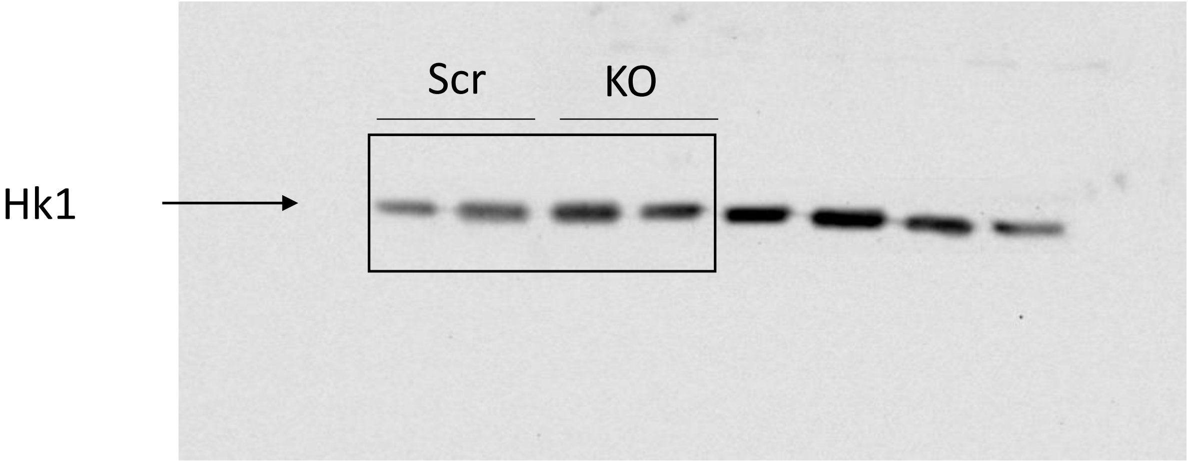

For Fig 4I

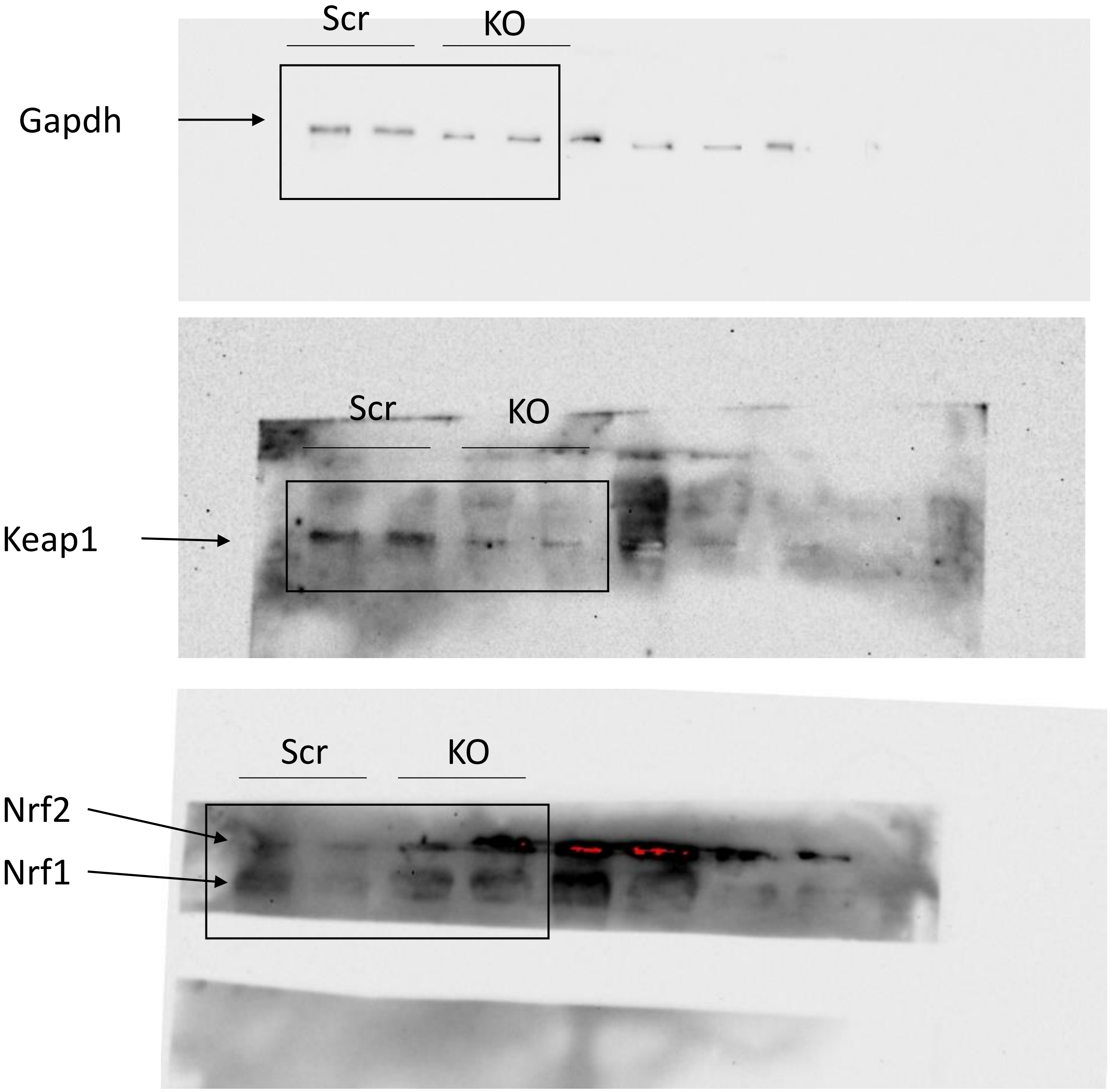

For Fig 4I

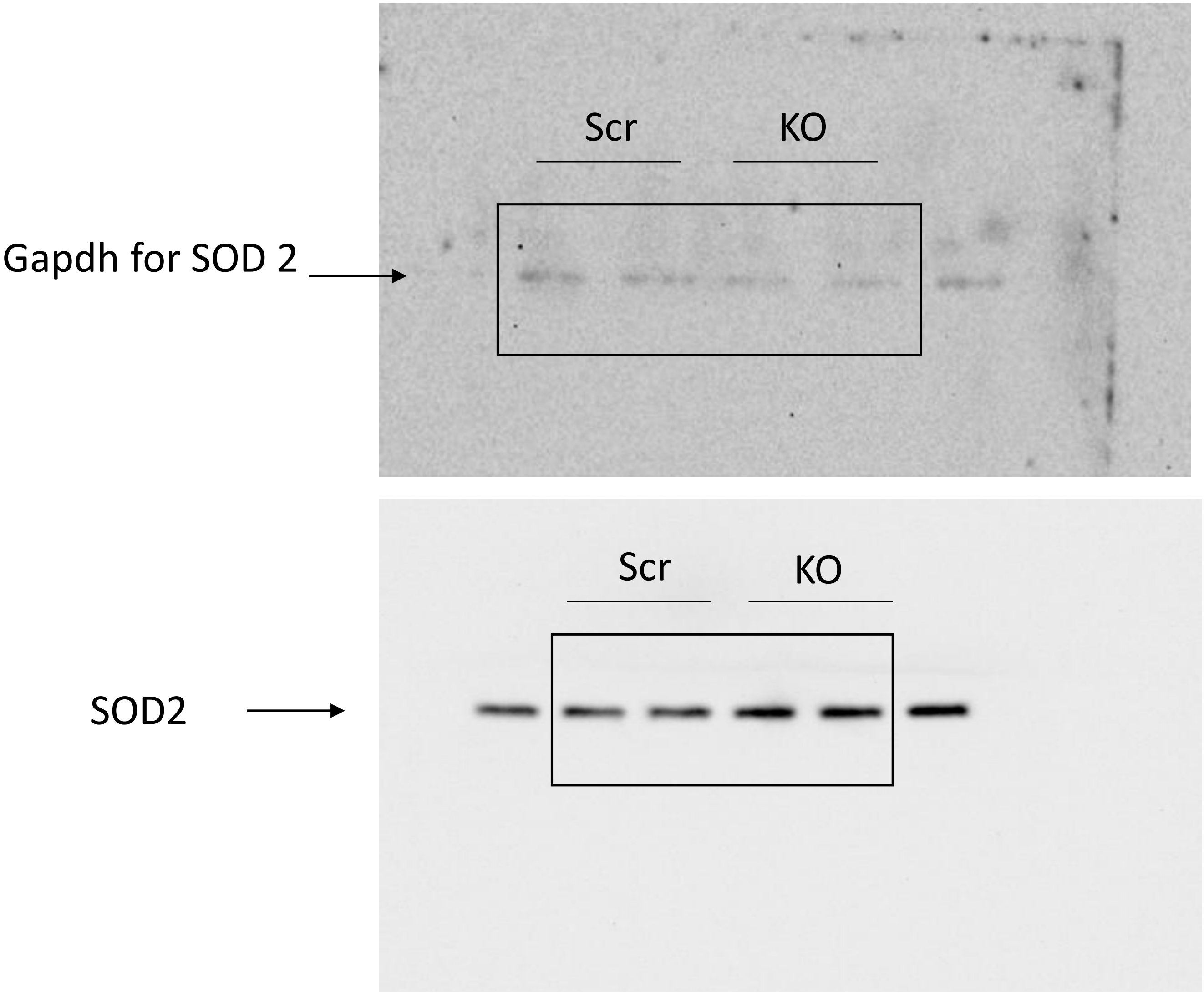

Supplement: Supplementary file 1 [file DataSheet1.zip › Data Sheet 2.PDF]
