# Supplementary material for: HMGB1 regulates mitochondrial structure and reactive oxygen species balance during the transition from naïve to primed pluripotency
Source: Front Cell Dev Biol. 2026 May 12;14:1807454. doi: 10.3389/fcell.2026.1807454 (PMC13201526; doi:10.3389/fcell.2026.1807454)
Supplement: Supplementary file 1 [file DataSheet1.zip › Figures S1-7 and Table S1.PDF]

Supplementary information

HMGB1 regulates mitochondrial structure and reactive oxygen species balance during the transition from naïve to primed pluripotency.

Tatiana Y. Starkova[1,*], Sergey V. Ponomartsev[1], Veniamin S. Fishman[2], Nariman R. Battulin[2], Nikolay D. Aksenov[1], Evgeny I. Bakhmet[1], Andrey A. Kuzmin[1], Dmitry S. Bogolyubov[1], Sergey A. Sinenko[1,*], Alexey N. Tomilin[1,*]

[1]Institute of Cytology of the Russian Academy of Sciences, Laboratory of Molecular Biology of Stem Cells, 194064 St. Petersburg, Tikhoretsky Av. 4, Russian Federation

[2] Novosibirsk State University; Laboratory of Structural and Functional Organization of the Genome, 6300901, Novosibirsk, Pirogova str. 1, Russian Federation

*Corresponding authors: t.starkova@incras.ru, s.sinenko@incras.ru, a.tomilin@incras.ru

Table S1. Antibodies

№	antibody	Cat_№	Company
1	Anti -HMGB1	ab79823	Abcam
2	Anti -HmgB2	ab67283	Abcam
3	Anti -actin	JLA20-s	DSHB
4	Anti -Gapdh	14C10	Cell Sign
5	Anti -Nanog	A300-397A	Bethyl
6	Anti -Oct4	C10	Santa Cruz
7	Anti -Tju1	MMS435P	Covance
8	Anti -Brachyury	AF2085	R&D Systems
9	Anti -Catalase	A11220	Abclonal
10	Anti -Sod2	A19576	Abclonal
11	Anti -Aldoa	A11445	Abclonal
12	Anti –Ldhb	A5131	Abclonal
13	Anti -HK1	A0533	Abclonal
14	Anti -NRF1	A3252	Abclonal
15	Anti -NDUFS1	PA5-22309	invitrogen
16	Anti -NDUFB10	MA5-26082	invitrogen
17	Anti -Keap1	GB113747	Servecebio
18	Anti -NRF2	GB113808	Servecebio
19	Anti –Cox4	A11631	Abclonal
20	Anti –Qcr2	A4366	Abclonal
21	Peroxidase AffiniPure F(ab')$_2$ Fragment Goat Anti-Rabbit IgG (H+L)	111-036-045	Jackson Immuno Research
22	Peroxidase AffiniPure F(ab')$_2$ Fragment Goat Anti-Mouse IgG (H+L)	115-036-062	Jackson Immuno Research
23	Cy™3 AffiniPure Goat Anti-Rabbit IgG (H+L)	111-165-144	Jackson Immuno Research
24	Alexa Fluor® 488 AffiniPure Goat Anti-Mouse IgG (H+L)	115-545-146	Jackson Immuno Research
25	Alexa Fluor® 647 AffiniPure Goat Anti-Mouse IgG (H+L)	115-605-003	Jackson Immuno Research
26	Alexa Fluor® 488 AffiniPure Goat Anti-Rabbit IgG (H+L)	111-545-144	Jackson Immuno Research
27	Anti-Oct6	Ab272925	Abcam

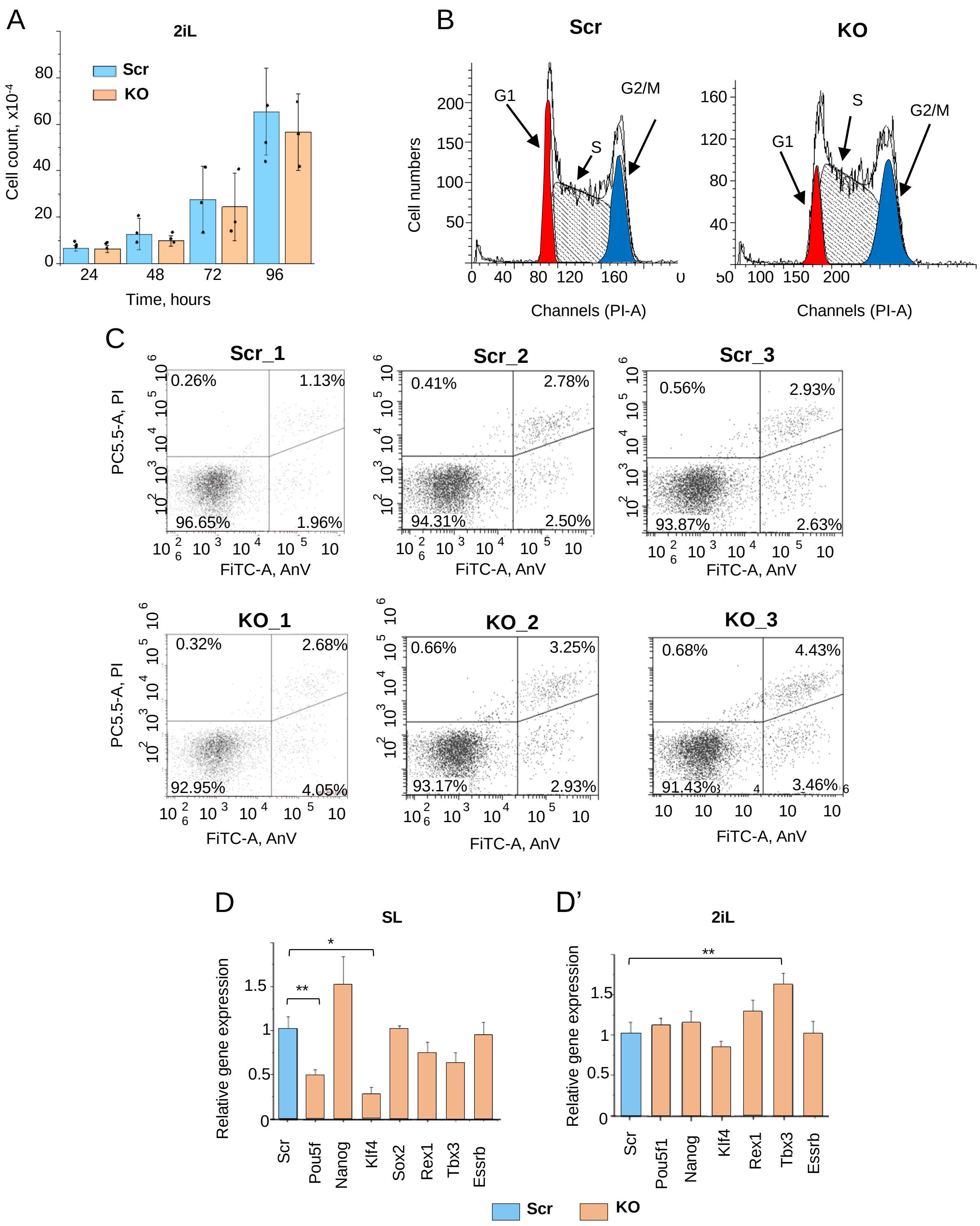

Figure S1. Loss of HMGB1 function does not affect ESC self-renewal.

(A) Growth curves determined by FACS analysis revealed comparable proliferation rates of Scr and KO ESCs in 2iL media, means ± SD, n=3 biological replicates. Time intervals are indicated in hours.

(B) Representative cell cycle phase distribution profiles of KO ESCs versus Scr controls, as determined by FACS analysis, means ± SD, n=3 biological replicates.

(C) Proportions of apoptotic and necrotic cells KO cells compared to Scr controls, as determined by Annexin V/propidium iodide staining and FACS analysis, n=3 biological replicates.

(D-D') RT-qRCR analysis illustrating relative expression of pluripotency genes in KO ESCs compared to Scr controls under SL and 2iL culture conditions, means ± SD, n=3 biological replicates *$p < 0.02$, **$p < 0.05$

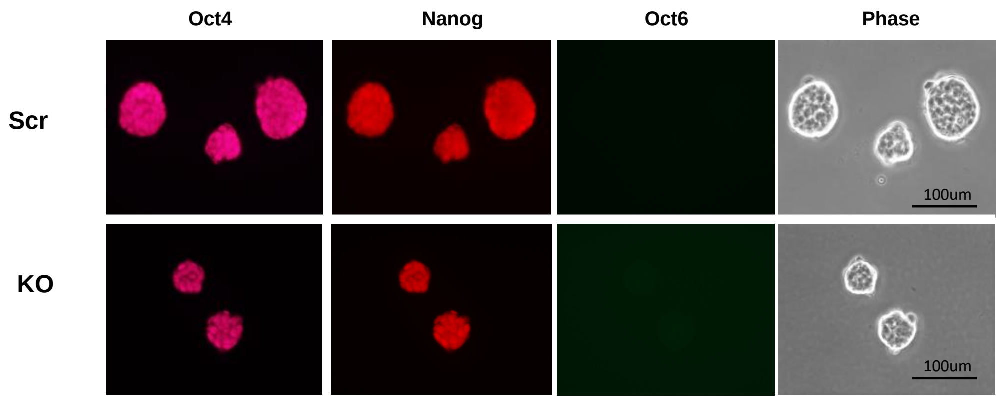

Figure S2. **Morphology and TF expression in KO ESCs under serum-free 2iL culture conditions at day 0 of in vitro differentiation toward EpiSCs.** Immunocytochemistry confirms proper expression of Oct4 (violet), Nanog (red), with absence of Oct6 (green) in both KO and Scr control ESCs. Phase-contrast images (Phase) show colony morphology.

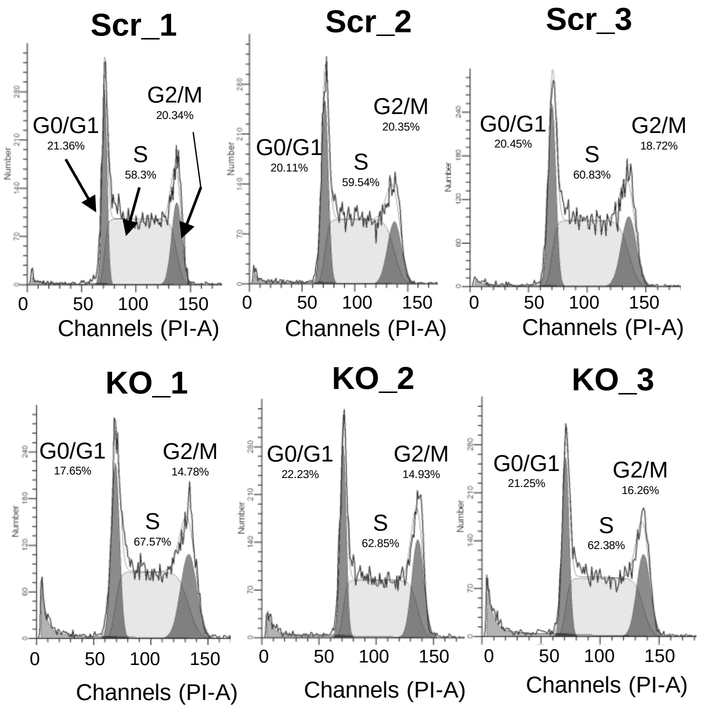

Figure S3. Altered cell cycle phase progression of KO cells during the naïve-to-primed pluripotency transition.

FACS profiles of KO and Scr control cells at day 3 of differentiation toward EpiSCs, showing altered cell cycle phase distribution in KO cells.

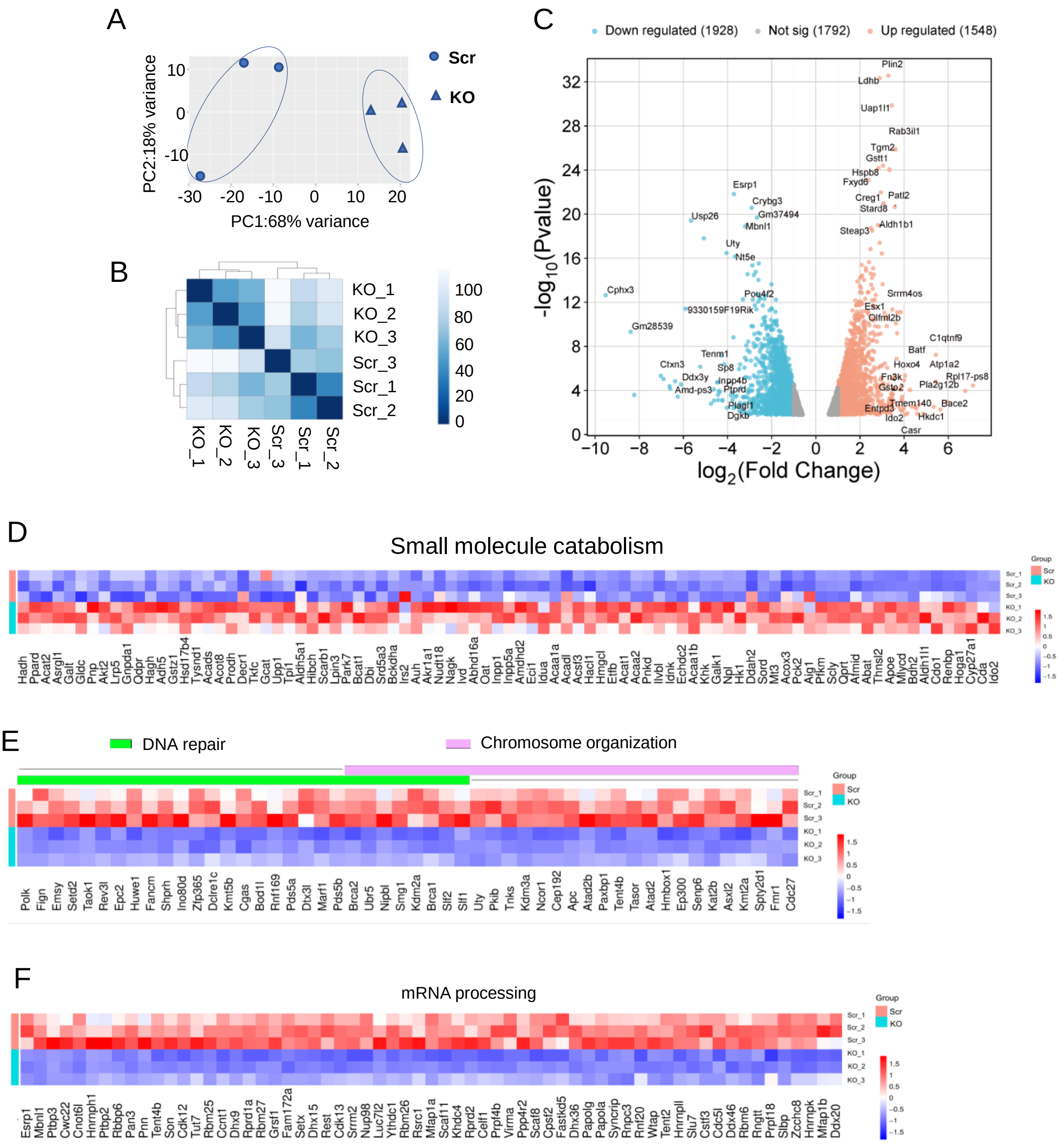

Figure S4. Transcriptome analysis reveals upregulated small molecule catabolism genes and downregulated chromatin-associated genes in KO ESCs.

(A) Principal component analysis of transcriptome profiles.

(B) Correlation matrix of gene expression between samples.

(C) Volcano plot identifying 1548 upregulated and 1928 downregulated genes in KO versus Scr ESCs.

(D-F) RNA-seq heatmaps generated with SRplot, showing downregulated DEGs associated with: (D) small molecule catabolism processes, (E) DNA repair and chromosome organization and (F) mRNA processing.

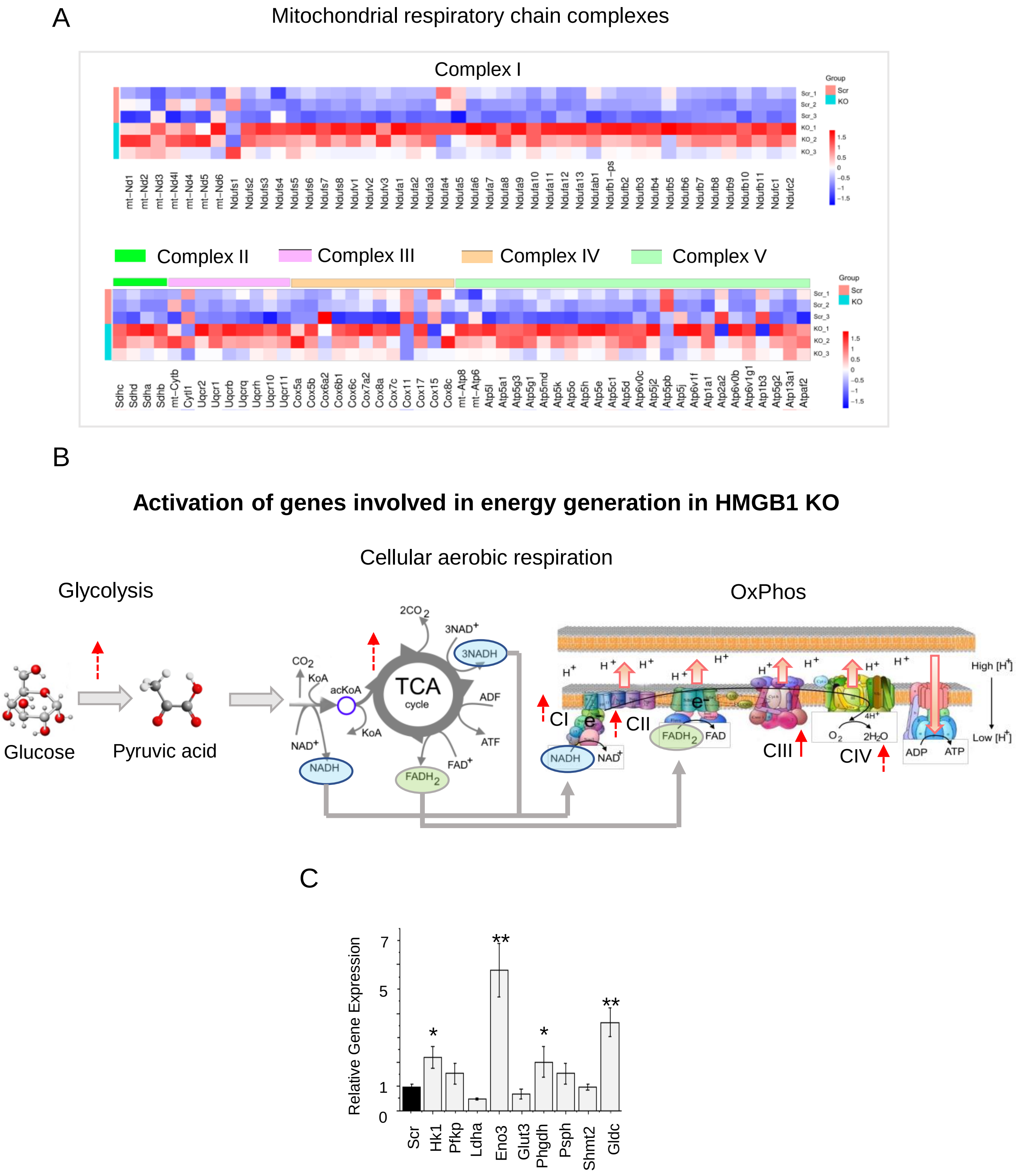

Figure S5. Transcriptome analysis reveals upregulated energy metabolism genes in KO ESCs. (A) RNA-seq heatmaps generated with SRplot, showing upregulated DEGs associated with electron transport chain complexes. (B) Schema of activation of gene expression associated with energy metabolic processes in KO ESCs. (C) RT-qRCR analysis illustrating significant upregulation of Hk1, Eno3, Phgd and Gldc gene expression in KO ESCs compared to Scr controls. Data are presented as mean ± SD, n=3 biological replicates, $*p < 0.05$, $**p < 0.01$

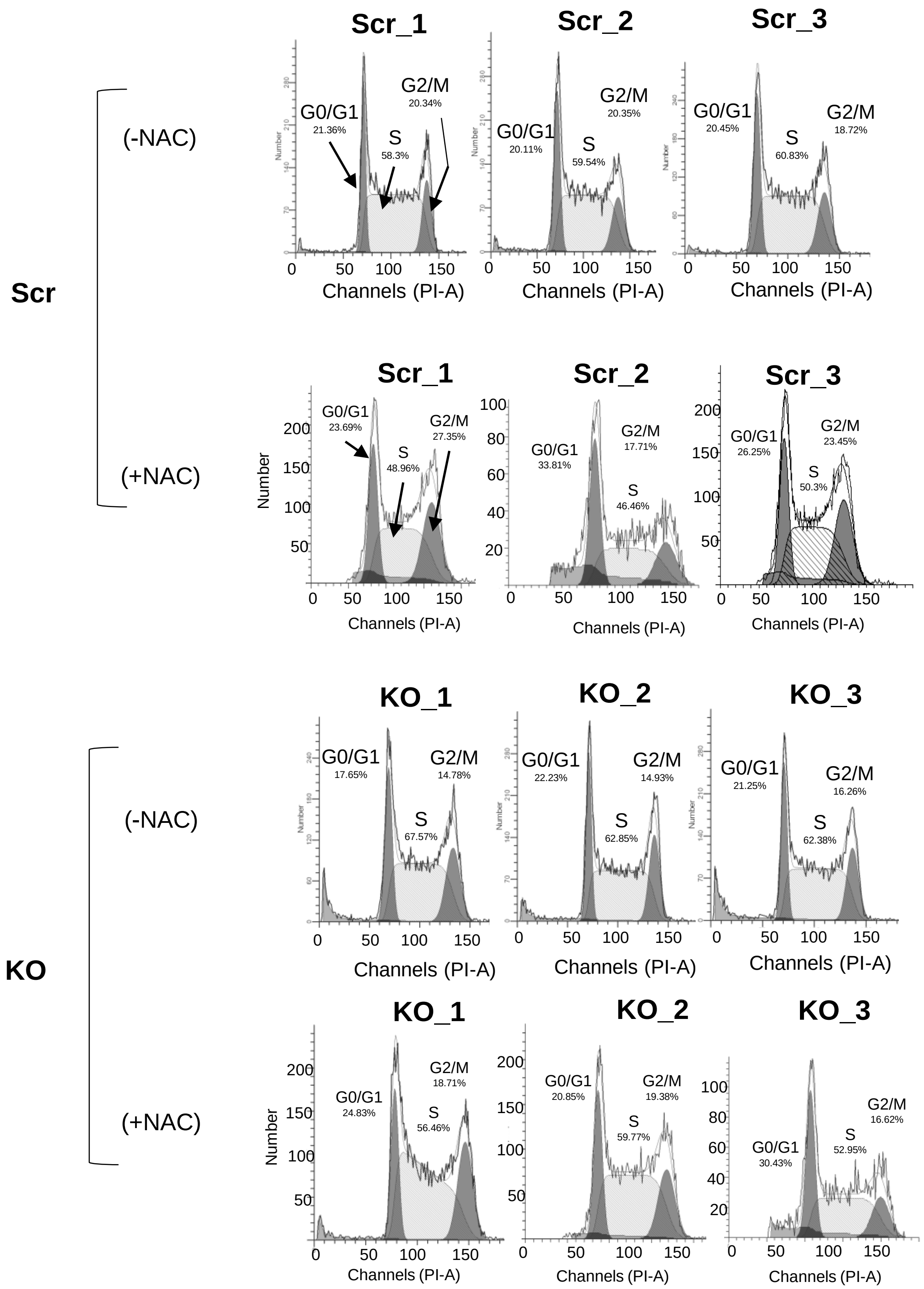

Figure S6. Rescue of impaired cell cycle progression by NAC treatment of KO cells during the naïve-to-primed pluripotency transition.

FACS profiles of KO cells, untreated versus 2.5 mM NAC-treated, at day 3 of differentiation toward EpiSCs, showing restored cell cycle phase distribution with treatment; n=3 biological replicates.

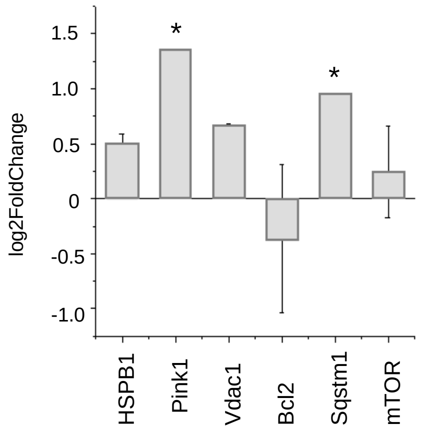

Figure S7. RT-qRCR analysis showing significant upregulation of Pink1 and Sqstm1 gene expression in KO cells compered to Scr. Data are presented as mean ± SD, n=3 biological replicates, *p < 0.05
